# Estimating the sensitivity and specificity of serum ELISA and pooled and individual fecal PCR for detecting *Mycobacterium avium* subspecies *paratuberculosis* in Canadian cow-calf herds using Bayesian latent class models

**DOI:** 10.3389/fvets.2022.937141

**Published:** 2022-07-29

**Authors:** Paisley Johnson, Lianne McLeod, John Campbell, Marjolaine Rousseau, Kathy Larson, Cheryl Waldner

**Affiliations:** ^1^Department of Large Animal Clinical Sciences, Western College of Veterinary Medicine, Saskatoon, SK, Canada; ^2^Département de Sciences Cliniques, Faculté de Médecine Vétérinaire, Université de Montréal, Saint-Hyacinthe, QC, Canada; ^3^Department of Agricultural and Resource Economics, College of Agriculture and Bioresources, Saskatoon, SK, Canada

**Keywords:** Johne's disease, beef cattle, prevalence, sensitivity, specificity

## Abstract

While Johne's disease (JD) is less common in beef than in dairy herds, consolidation is increasing transmission risk. Estimates of *Mycobacterium avium* spp. *paratuberculosis* (MAP) prevalence and test performance in cow-calf herds are needed to inform control programs. Objectives of this study included describing the prevalence of MAP in Canadian cow-calf herds and comparing the relative performance of a serum ELISA, pooled fecal PCR and individual fecal PCR using Bayesian latent class models, and to investigate factors associated with positive MAP tests. Blood and fecal samples (*n* = 3,171) were collected from 159 Canadian cow-calf herds. All samples were analyzed using serum ELISA and fecal PCR (pools of five samples) and a subset of 913 fecal samples were also tested with individual PCR. Based on latent class analysis, MAP prevalence was higher in eastern compared to western Canada for both animals {East, 3% [95% Credible Interval (CrI) 1–7%]; West, 1% [95% CrI 0.2–2%]} and herds [East, 15% (95% CrI 2–35%); West, 10% (95% CrI 1–26%), based on one or more positive results]. Sensitivity (Se) and specificity (Sp) for animal level individual PCR were 96% (95% CrI 80–100%) and 98% (95% CrI 96–100%), respectively followed by pooled PCR [Se = 54% (95% CrI 36–72%), Sp > 99.9% (95% CrI 99.8–100%)] and ELISA [Se = 36% (95% CrI 22–52%), Sp = 98% (95% CrI 96–99%)]. Based on 20 samples per herd, the herd level Se of ELISA was 79% (95% CrI 47–100%) (at least one positive sample) compared to 43% (95% CrI 14–94%) for pooled PCR. Herd-level Sp was 99% (95% CrI 96–100%) for pooled PCR and 90% (95% CrI 83–100%) for ELISA. Cows from herds with dairy cattle on farm and cows with symptoms of JD in the past 3 years were more likely to be MAP positive. Herds that had animals with JD symptoms in the previous 3 years and those with more breeding females were most likely to test positive for MAP. While serum ELISA can be effective for herd screening, PCR performed better for animal testing. Pooled PCR testing could be a less costly option; however, determining the most cost-effective approach will require further economic analysis.

## Introduction

Johne's disease (JD) is a form of chronic enteritis in domestic ruminants characterized by profuse diarrhea and emaciation resulting in death (1). The causative agent of JD is the gram-positive bacterium *Mycobacterium avium subspecies paratuberculosis* (MAP) (2). Johne's disease has a prolonged incubation period ranging from 2 to 5 years during which infected animals progress through four stages of disease (3, 4). The silent (infected but not shedding MAP), subclinical (no clinical signs but shedding MAP), clinical and advanced clinical stages of infection are defined by the likelihood of detecting MAP in the feces or MAP antibodies in the blood and the emergence of clinical signs (3). There is no treatment, and while vaccines are available in some countries (5) there is currently no licensed vaccine available for use in Canada (6).

The estimated prevalence of MAP within Canadian beef herds is low at <1–2% (7, 8). However, consolidation of the beef industry into fewer, larger herds could result in an increase in the prevalence of MAP as was observed in the dairy industry (9, 10). Johne's disease is difficult to control (5) and poses a substantial threat to the beef industry due to impacts to animal health, welfare and productivity in affected herds (11). Current information is needed to inform prevention and control measures.

There is no gold standard test for identifying animals infected with MAP. Available diagnostic tests typically have limited sensitivity and moderate to high specificity; however, these estimates vary based on stage of infection (12). Infected animals in the silent stages of infection typically do not shed MAP in their feces. Those in the subclinical stage of disease might not shed sufficient levels of MAP in their feces or have sufficient serum antibody levels to reach the threshold of detection by the fecal polymerase chain reaction (PCR) test or serum enzyme-linked immunosorbent assay (ELISA) (3, 13, 14). Imperfect test performance coupled with the delayed onset of clinical signs make it difficult to accurately identify infected animals for disease control and for estimating true disease prevalence.

Several previous studies have used fecal culture as a gold standard reference test to estimate the sensitivity and specificity of another diagnostic test of interest (13, 15–20). However, fecal culture does not have a perfect sensitivity or specificity, and comparison to an imperfect gold standard can generate biased results (21). Bayesian latent class models (BLCMs) provide an alternative method for estimating diagnostic test sensitivity and specificity as well as disease prevalence in the absence of a gold standard. Latent models facilitating the cross comparison of two diagnostic tests in two populations were first described by Hui and Walter (22). Implementation of latent class models in a Bayesian framework has evolved from this paradigm and allow estimation of diagnostic test accuracy for two or more tests in one or more populations (21).

Previous estimates of diagnostic test performance for detecting MAP using a BLCM approach have been reported from eastern Canada, New Zealand, Chile and the U.S., but have focused primarily on dairy cattle (23–29). While data from the dairy industry is helpful, having test performance data specific to the beef industry is necessary due to the vastly different management and current risk of infection for these two commodities. In Canada as well as many other regions with large scale cow-calf production, cow-calf herds are typically extensively managed outdoors while dairy cows are more intensively managed. The resulting risk of calf exposure to MAP as well as the opportunities for disease management can be very different between beef and dairy herds (30).

With more understanding of diagnostic test performance in beef herds in addition to current prevalence data, veterinarians can better inform testing strategies and JD control programs. Whole herd testing is costly and time consuming particularly for large commercial operations. Information on herd and animal factors associated with JD that could be used to target risk-based testing programs in beef herds is also limited (31–34). With additional evidence, veterinarians could more effectively identify herds at greatest risk of infection and potentially animals within those herds most likely to present a transmission threat.

The primary objective of this study was to describe the prevalence of MAP in Canadian cow-calf herds based on testing serum and pooled fecal samples from herds enrolled in a national surveillance program. The second objective was to compare the relative performance of a serum ELISA, pooled fecal PCR and individual fecal PCR for identifying positive herds and positive animals within those herds using BLCMs. The third objective was to describe factors associated with herds and cows most likely to test positive to either serum ELISA or the pooled fecal PCR.

## Materials and methods

### Description of eligible study population

Participants were enrolled from the Canadian Cow-Calf Surveillance Network (C^3^SN). The purpose of the C^3^SN was to estimate the prevalence of production-limiting diseases in beef herds across Canada to improve herd health and productivity. One hundred and eighty-one producers were initially recruited to the network in mid to late 2018 through veterinarians, social media, provincial beef associations and fellow producers. Criteria for recruitment included operations that conducted pregnancy checking, had greater than 40 breeding animals and access to email. Baseline information was collected for each herd at the time of enrollment in the C^3^SN. Of the 181 producers that were initially enrolled, 178 provided complete baseline information of which 176 identified they were also willing to participate in blood and fecal sample collection as part of a sample banking project for infectious disease and trace mineral studies.

### Sample collection

This study was based on a cross-sectional sample and data collection from volunteer herds participating in C^3^SN; consequently, prevalence estimates may not be generalizable to the wider Canadian beef herd. Completion of the analysis of the blood and fecal samples for MAP was contingent on available funding and the herd owners and veterinarians were not told that MAP analysis would be completed at the time of recruitment, rather permission was obtained to test collected samples more generally for markers of infectious disease and micronutrient status. Sample size was not planned for this analysis, rather all available samples were included in the initial screening.

Blood and fecal samples were collected in the fall of 2019 at the same time as pregnancy testing by private veterinarians selected by the herd owners. Veterinarians were instructed to collect a systematic random sample of 20 cows in each herd regardless of the herds' sizes (for example, every fifth cow in a herd of 100 cows). Information was collected on age and body condition score (BCS) of each cow sampled at the time of testing. Information on MAP clinical status of the cows was not collected. Following collection, whole blood and fecal samples were sent to a diagnostic laboratory in insulated coolers *via* overnight courier (shipping time 1–4 days depending on point of origin) for processing and analysis. Testing results were provided by the diagnostic lab directly to the submitting veterinarians.

### Sample processing strategy

**Figure 1:**
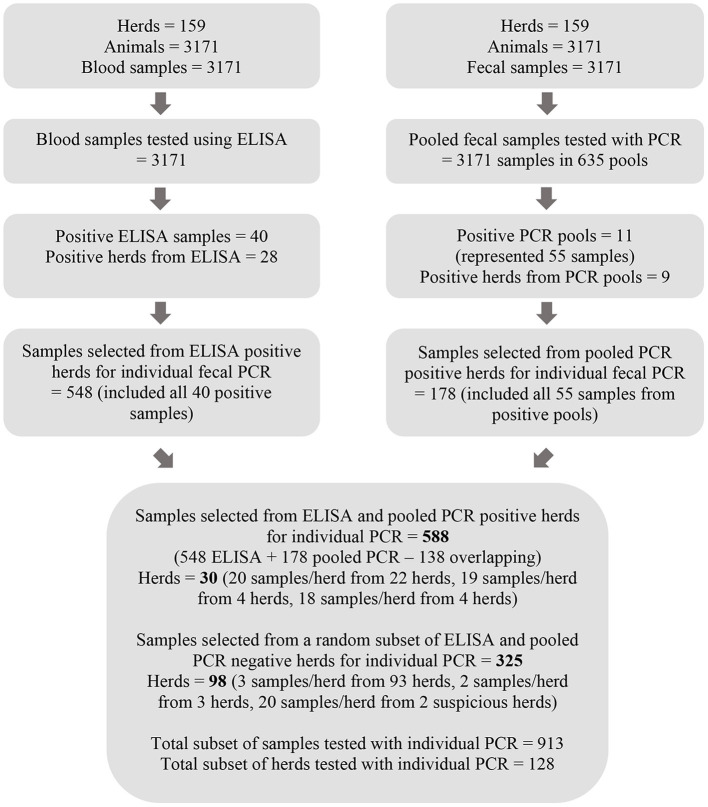
Flow chart depicting the process for testing serum samples with MAP ELISA and pools of five fecal samples with PCR as well as the method by which a subset of fecal samples was selected for further testing with individual PCR.

All serum samples were analyzed individually using ELISA and all fecal samples in pools of five using PCR followed by individual PCR on samples within positive pools. A subset of samples as determined by ELISA and pooled PCR results were also selected for individual fecal sample PCR testing (Figure 1). All available fecal samples from herds with either a positive pooled PCR result or a positive ELISA result were eligible for testing with individual PCR. A random subset of 3 samples per herd from herds where there were only negative test results from both ELISA and pooled PCR were also eligible for further testing. Twenty samples were selected from two herds with suspicious ELISA test results defined by a sample to positive ratio of 0.45 < S/P < 0.55. All eligible fecal samples were individually tested with PCR given the volume of the remaining stored sample was sufficient.

### Sample analysis

All samples were analyzed by the regional commercial laboratory [Prairie Diagnostic Services (PDS) Inc., Saskatoon, Saskatchewan, Canada]. The PDS laboratory is accredited by the American Association of Veterinary Laboratory Diagnosticians that follows ISO 17025 standard for testing. The lab is also accredited by the Standards Council of Canada for a number of tests including both real time PCR and ELISA assays reported in this study.

The technicians in different areas of the lab completing the various tests were blind to corresponding blood and fecal sample results and unaware of plans to do a comparative analysis. The lab did not have access to any cow or herd level data other than the identification of the submitting veterinarian.

The tests chosen for comparison were the test choices provided to veterinarians by the regional commercial laboratory and used by veterinarians to diagnose JD in beef herds (35), and the tests most commonly reported as part of regional surveillance programs (36).

### Serum samples

Clotted blood samples were centrifuged by the laboratory and serum was aliquoted and stored at −80°C until processing. All serum samples (*n* = 3,171) were analyzed individually using a commercially available MAP-specific ELISA test (*Mycobacterium paratuberculosis* Antibody Test Kit, IDEXX Laboratories, Westbrooke, Maine, USA) according to the manufacturer's recommendations. Samples were determined to be positive, suspect or negative if the sample to positive (S/P) ratio was ≥ 0.55, 0.45 < S/P < 0.55 or S/P ≤ 0.45, respectively using the IDEXX XChekPlus® software as per the test manufacturer's recommendations.

### Pooled fecal samples

All fecal samples were analyzed using the VetAlert™ Johne's Real-Time PCR kit (Tetracore Inc., Rockville, MD, US) which targets the hspX gene of MAP and is commonly used in North American diagnostic laboratories. Samples were processed in pools of five where possible (*n* = 631) with remaining samples processed as pools of four (*n* = 1), three (*n* = 2) and two (*n* = 1) animals for DNA extraction following the “Pooling Bovine Fecal Samples” section of the manufacturer's protocol for the Tetracore MAP Extraction System (Tetracore Inc., Rockville, MD, US). After transferring 2g fecal material into 50 ml sterile plastic conical tubes and adding 35 ml 1× Tetracore Extraction (TE) buffer, samples were treated as described in the individual protocol. Afterwards, pools of samples were prepared by combining 4 ml of supernatant of 5 different individual samples in one 50 ml conical tube, for a final volume of 20 ml. The remaining 20 ml of supernatant from each sample was stored at −20°C in case further individual testing was required. Subsequent steps were the same as described for individual samples below. Suspect pools were re-tested and each sample from the positive pools was tested following the individual sample protocol. Samples from positive pools were reported as per their subsequent individual test results. Samples from negative pools were reported as negative.

### Individual fecal samples

A subset of fecal samples (*N* = 913) selected as described in Figure 1 were processed individually for DNA extraction using the Tetracore MAP Extraction System following the manufacturer's instructions from “Two Gram Protocol: for Maximum Sensitivity.”

Extracted DNA from fecal samples was stored at −20°C and subsequently analyzed using the VetAlert™ Johne's Real-Time PCR kit using the Bio-Rad CFX96 Touch Real-Time PCR Detection System (Bio-Rad Laboratories Inc., Hercules, CA, USA). Samples were considered positive if the Ct was ≤ 38, and suspect if the samples crossed the threshold after the positive cut-off value as per the manufacturer's recommendations.

### Data management and statistical analyses

Baseline data on the study herds were collected using a hard copy mailed survey and included type of operation, production activities and selected management practices. The baseline surveys had been sent in late December 2018 and were collected during 2019. Follow-up questions regarding history of JD and purchasing replacement animals were sent *via* email in 2021. This information was linked to animal identification, age and body condition score (BCS) data collected at the time of testing. Herds were categorized into either the western region (Saskatchewan, Alberta, British Columbia and Manitoba) or the eastern region (Atlantic provinces, Ontario and Quebec).

For the purposes of this analysis, infection with MAP was considered to be a condition where entrance and persistence of MAP elicits an immune response to MAP detectable by ELISA or results in shedding of MAP nucleic acid detectable by PCR in the feces; the target condition included any stage of infection with MAP.

Test results for individual animals and for study herds were summarized for ELISA, pooled fecal PCR and individual animal fecal PCR results. Animals in a PCR positive pool were considered positive if they were positive when individually retested as per manufacturer's protocol. Suspicious test results were categorized with negative test results. Two-by-two and two-by-two-by-two contingency tables were generated for all combinations of test comparisons for both animal and herd level data. For analysis at the pool level, pooled PCR test results and ELISA test results were also summarized by pool ID to compare the pools positive by PCR to the pools with at least one positive ELISA.

For the herd level analysis, positive herds were defined by one or more positive PCR sample(s) for both pooled and individual protocols. Herd-level ELISA data were analyzed using two cut-off values: one or more positive sample per herd as well as two or more positive samples per herd. The two-sample cut-off for defining ELISA positive herds has been reported in previous studies to increase specificity at the herd level (33).

Kappa statistics were generated using publicly available software (37) to determine the agreement beyond chance for all possible pairings of the diagnostic tests under study at the individual animal and herd level (38).

### Bayesian latent class models

Bayesian latent class models were developed to estimate diagnostic test sensitivity and specificity in the absence of a gold standard using Markov Chain Monte Carlo (MCMC) methods. Use of these models requires the following assumptions: the diagnostic tests should be independent, the prevalence should vary between the target populations, and the sensitivity and specificity of the tests under evaluation should be constant across target populations (39). Guidelines for reporting studies of diagnostic test accuracy using BLCMs (40) and for paratuberculosis in ruminants (41) were followed. Initially a two-test (pooled PCR and ELISA) and two-population (western and eastern regions) model was constructed for the full data set at both the individual and herd level. The two test, two population models estimate the sensitivity and specificity of each test, and the true disease prevalence in both populations yielding a fully identifiable model with 6 parameters and 6 degrees of freedom, with degrees of freedom equal to (2^*R*^ – 1)*S* for *R* tests applied to *S* populations (22).

In a second step, three-test (pooled PCR, individual PCR and ELISA), two-population (west and east) models were developed for the subset of samples with individual PCR at the animal level to extend the analysis to individual PCR and optimize the estimations of sensitivity and specificity. As this was not a random subset of the population, resulting estimates of prevalence were not considered meaningful. A covariance term was added to this model to address conditional dependence between pooled PCR and individual PCR (42, 43) while ELISA and PCR (pooled and individual) were assumed to be independent because these tests are based on the detection of different biological markers from different samples. The three test, two population models estimate the sensitivity and specificity of each test, the covariance between individual and pooled PCR as well as the disease prevalence in both sample subsets yielding a fully identifiable model with 10 parameters and 14 degrees of freedom.

A series of additional two-test, two-population models were developed for the subset of samples with complete data on all three tests at both the animal and herd level for comparison. A final two-test, two population model at the pool level was also developed to examine sensitivity and specificity for the detection of MAP with PCR in the pools of five samples in comparison to whether any animals within the pools were positive based on ELISA. Sample code is included in the Supplementary Material.

The models were developed and run using JAGS software (44) and the runjags package (45) in R (R Foundation for Statistical Computing, Vienna, Austria). Non-informative prior distributions [beta(1, 1)] for sensitivity, specificity and prevalence were used in models for the primary analysis. Parameters for models were estimated using 250,000 iterations of 3 chains after a burn-in adaption phase of 50,000 iterations.

Convergence diagnostics included in runjags summary statistics [potential scale reduction factor (46), Monte Carlo standard errors (47), effective sample size] and visual inspection of trace and autocorrelation plots were used to evaluate convergence. Estimates for test sensitivity, test specificity, and prevalence were reported as the median of the posterior along with 95% credible intervals (95% CrI). Posterior distributions from each model were compared using the overlapping package in R (48). The overlap index represents the proportion of overlap between distributions normalized between 0 and 1, where 0 reflects completely separate and 1 reflects completely overlapping distributions; no assumptions are required about distributional form (49). The distribution of numeric differences between the posterior chains were also evaluated.

### Informative priors and sensitivity analysis

Informative priors were developed using test sensitivity and specificity data from peer-reviewed published studies reporting sensitivity and specificity estimates for the same diagnostic tests used in this study and from animals that were considered subclinical. Four studies were identified that reported a sensitivity estimate for ELISA (IDEXX) (50–53) and three of those studies also reported specificity estimates (50–52). Parameters for the informative beta prior distributions were calculated from the literature as follows: alpha = *x*+1 and beta = *n* – *x* + 1 where x was the number of successes and n was the number of tests summed across the relevant studies for each type of test (Table A in Supplementary Material). The resultant prior distributions were beta (187,391) for ELISA sensitivity and beta (65,122) for ELISA specificity. Only one study reported a sensitivity and specificity estimate for individual PCR using the Real-time PCR—Tetracore VetAlertTM kit (54) while no studies were identified that reported estimates for pooled PCR using this kit. Estimates from this study were used to develop priors using the EpiR beta buster function (55); beta (48.33, 18.51) and beta (27.71, 2.11) distributions were used for both individual and pooled PCR sensitivity and specificity, respectively (Table A in Supplementary Material).

Sensitivity of the models to the choice of priors was evaluated by comparing posterior estimates from models using uninformative [beta (1, 1)] priors to models using the informative priors for all tests. This sensitivity analysis using informative priors was applied only to the three test comparisons for individual data as informative priors for herd level analysis were not available and herd level results would be expected to vary based on sample size.

### Multivariable regression to examine potential risk factors for MAP test positivity

Generalized estimating equations (GEE) using a logit link function and binomial distribution (StataCorp. 2021. *Stata Statistical Software: Release 17*. College Station, TX: StataCorp LLC.) were used to determine associations between JD risk factors and animal and herd positivity status while accounting for clustering of infection within herds with robust standard errors for the animal level analysis. The associations between each risk factor and ELISA or PCR positivity status were first analyzed using unconditional models. Risk factors considered for analysis were based on previously recognized risk factors for JD and available data included: age and BCS reported at pregnancy testing, geographical region, whether there was a confined calving location as compared to calving on pastures, month calving started, if dairy cattle were kept on-farm, if cows were grazed on communal pasture, and the number of females exposed to breeding. Further risk factors that were analyzed in a separate model for a subset of study herds for which data were available included having JD diagnosed within the herd by a veterinarian prior to sampling, having animals show symptoms of JD within the last 3 years and purchasing replacement animals within the last 5 years.

If the *p* < 0.2 for the association between risk factor and positivity status, the variable was considered in building the final multivariable models. Potential confounders were retained in the model regardless of significance if inclusion changed effect estimates of interest by >25%. Two-way interactions were examined if more than two variables were retained as significant risk factors in the final model (*p* < 0.05) and the interaction was considered to be biologically plausible. Estimates were reported as odds ratios (OR) with 95% confidence intervals (95%CI).

## Results

### Study herd characteristics

Overall, samples from 167 cow-calf herds were received for MAP testing. Eight of the 167 producers were excluded from the study because they withdrew from the study or did not complete the survey designed to collect general information about the operation. The resulting 159 herds were located in British Columbia (*N* = 8), Alberta (*N* = 48), Saskatchewan (*N* = 33), Manitoba (*N* = 21), Ontario (*N* = 23), Quebec (*N* = 22) and the Atlantic provinces (*N* = 4). Most of the study herds identified as primarily commercial cow-calf operations (≥60% commercial females) (Table 1). The average herd size in the west and east was 230 (SD, 209) and 176 (SD, 168) females, respectively. Most of the sampled cows were reported as greater than 3 years old (2,423/3,147) and having a BCS between 2.5 and 3.5 (2,137/3,162) based on a 5-point scale; BCS was <2.5/5 for 8.0% of cows (253/3,162). The average number of cows and heifers exposed to breeding in the study herds in 2019 was 199 (SD, 191) and 42 (SD, 52), respectively and the average number of calves born alive to cows and heifers was 170 (SD, 157) and 30 (SD, 35) respectively.

**Table 1:** Summary of baseline characteristics, management practices and JD history and risk factors for 159 study herds by region.

	**Percentage (Number) of herds**		
	**East** ** (N** = **49)**	**West** ** (N** = **110)**	**Total** ** (N** = **159)**
**Operation type**			
Mostly commercial (≥60%)	76% (37)	83% (91)	81% (128)
Mostly purebred (≥60%)	16% (8)	15% (16)	15% (24)
Half purebred/commercial	8% (4)	2% (3)	4% (7)
**Production activities^a^**			
Backgrounding	57% (28)	64% (70)	62% (98)
Stocker	27% (13)	32% (35)	30% (48)
Feedlot	24% (12)	8% (9)	13% (21)
Other	24% (12)	25% (27)	25% (39)
**Calving time**			
Winter	33% (16)	37% (41)	36% (57)
Spring	51% (25)	63% (69)	59% (94)
Summer/fall	16% (8)	0	5% (8)
**Calving location**			
Confined	84% (41)	82% (90)	82% (131)
Non-confined	16% (8)	18% (20)	18% (28)
**JD specific risk factors**			
Dairy cattle on-farm	8% (4)	2% (2)	4% (6)
Use of communal pastures	8% (4)	25% (27)	19% (31)
Purchased replacement animals in last 5 years^b^	82% (23/28)	63% (50/80)	68% (73/108)
**JD history^b^**			
Had animal(s) show JD symptoms in last 3 years	11% (3/28)	26% (21/80)	22% (24/108)
JD diagnosed within herd by veterinarian prior to testing in fall of 2019	14% (4/28)	18% (14/80)	17% (18/108)

a *Some producers selected more than one option*.

b *Data available for only 108 herds (N east = 28, N west = 80)*.

Herd owners reported other production activities including backgrounding, stocker operations, and feedlots (Table 1). The timing of calving varied with most herds starting in the spring (Mar-Apr) followed by winter (Dec-Feb) (Table 1). Some eastern herds also reported starting to calve in the summer and fall months (Table 1). Dairy cattle were present on <4% of herds, but were slightly more frequent in the east (Table 1). More operations from the west report sending at least some their cattle to communal grazing pastures in 2018 (Table 1). Most producers reported calving in confined locations such as small paddocks, corrals or barns (Table 1).

Previous JD diagnosis history as well as the purchase of replacement cows and heifers were available for 108 of the 159 study herds (Table 1). Most herds had purchased replacement animals in the last 5 years (Table 1). A higher proportion of herds in the west reported they had animals show clinical signs of JD in the last three years (Table 1). In ~1 in 6 herds, JD had been diagnosed by a veterinarian prior to testing in the fall of 2019.

### Summary of individual animal testing data

**Figure 2:**
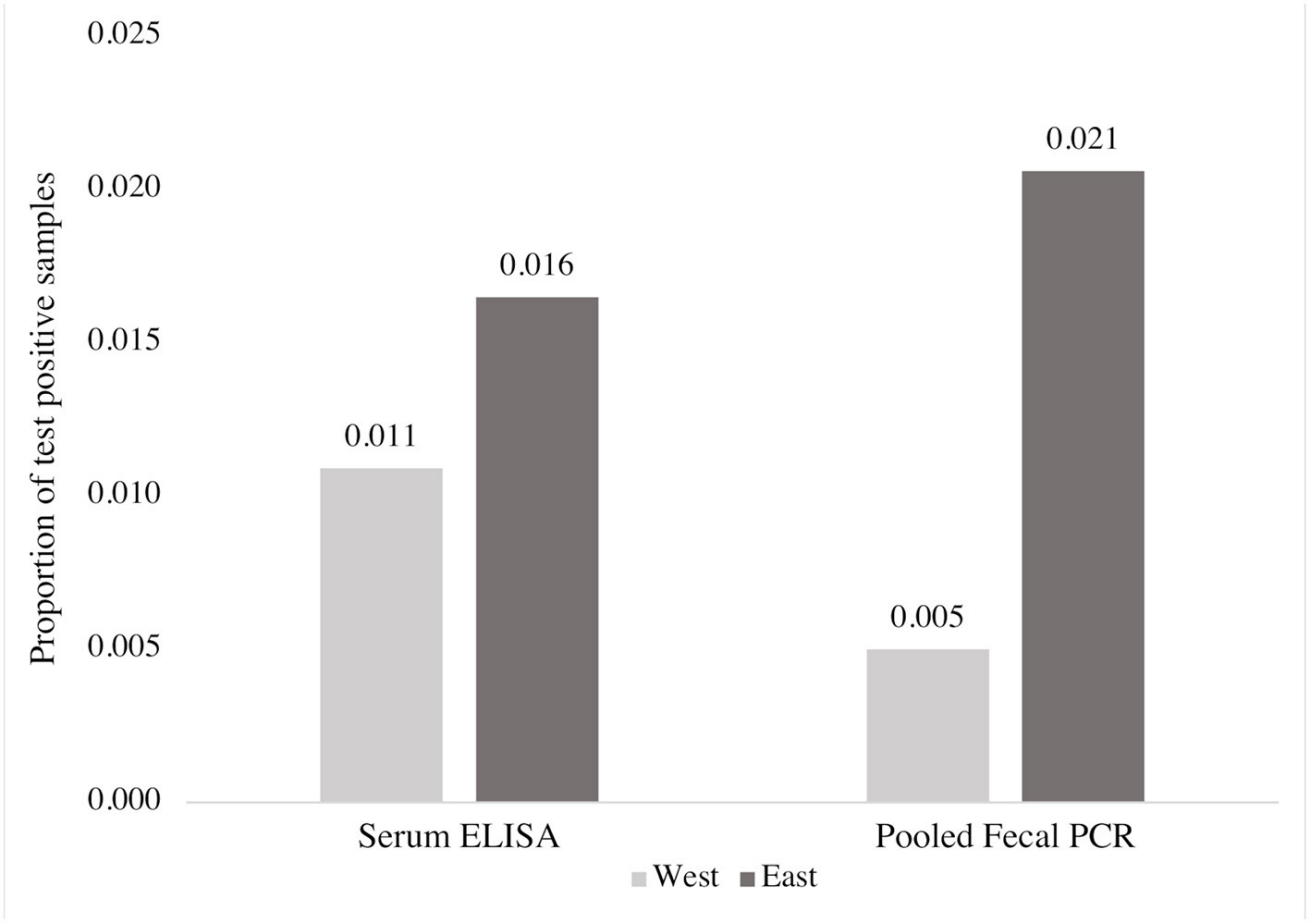
Comparison of the proportion of MAP positive blood and fecal samples detected by ELISA and fecal PCR (pools of five fecal samples), respectively, from 3,171 beef cows according to region.

Based on all individual cow samples (*n* = 3,171) collected using random sampling of participating herds, 1.3% (40/3,171) of cows in the study were positive for MAP on the ELISA test, and 0.9% (30/3,171) were positive by fecal PCR testing (pooled testing confirmed by individual testing). Both pooled PCR confirmed with individual PCR test results and ELISA detected a higher proportion of positive samples in the east compared to the west (Figure 2). Of the 3,171 samples, pooled PCR and ELISA agreed on the detection of 10 positive and 3,111 negative samples (kappa = 0.28) (Table 2).

**Table 2:** Comparison of MAP testing results for ELISA testing of serum samples as compared to PCR testing of pools of five fecal samples for 3,171 samples from beef cows examined with two diagnostic tests.

	**Pooled PCR**		
	**Positive**	**Negative**	**Total**
**ELISA**			
Positive	10	30	40
Negative	20	3,111	3,131
Total	30	3,141	3,171

*Kappa = 0.28*.

**Figure 3:**
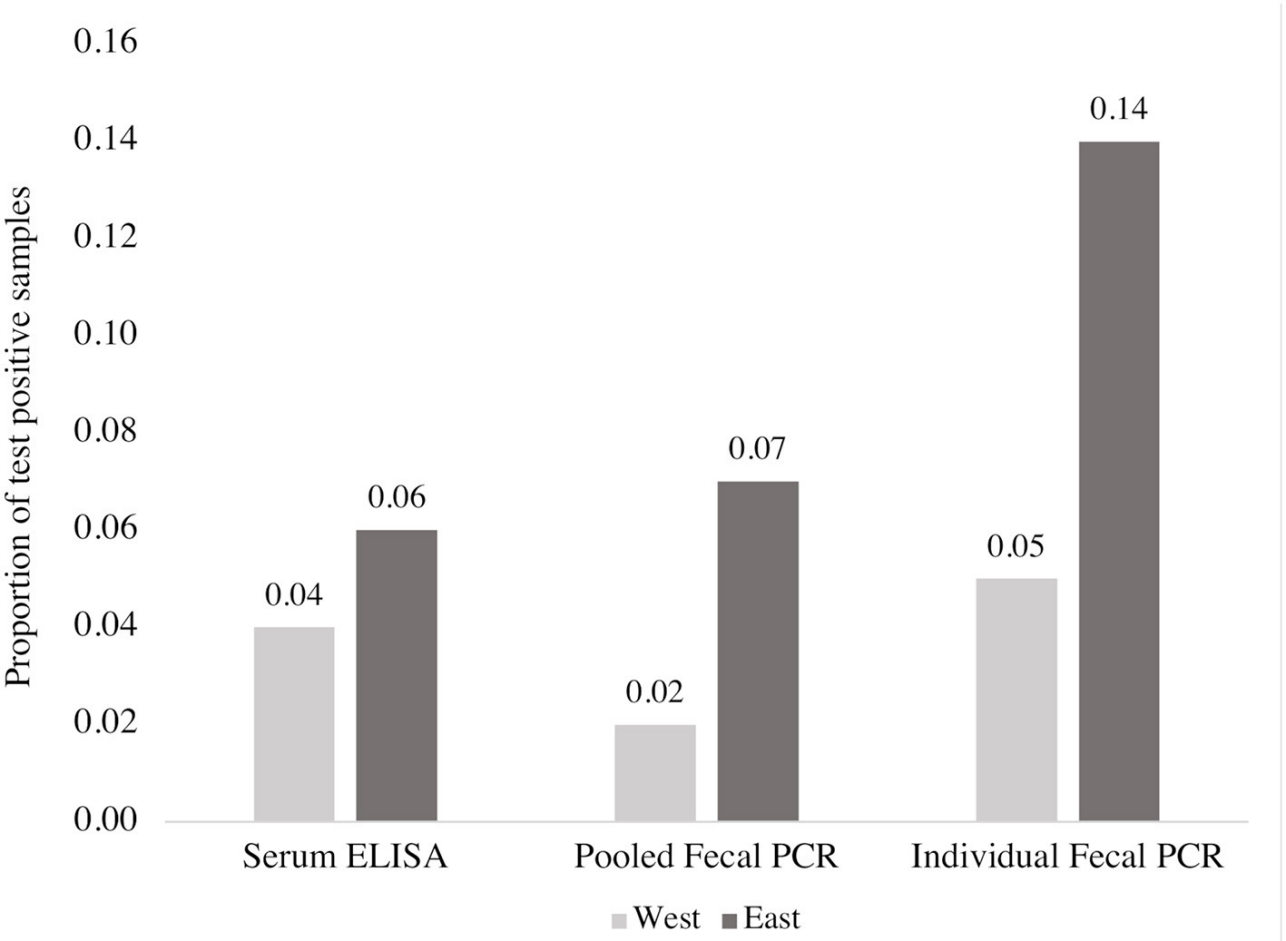
Comparison of the proportion of MAP positive blood and fecal samples detected by ELISA, fecal PCR (pools of five fecal samples) and individual fecal PCR from a subset of 913 beef cows according to region. This figure reflects data from a non-representative subsample of the overall data set (913 of 3,171 samples).

When the subset of animals tested with individual PCR (*n* = 913) were used to compare all three diagnostic tests, individual PCR detected the highest proportion of positive samples compared to ELISA and pooled PCR (Figure 3). Of the 913 samples that were also tested with individual PCR, ELISA and pooled PCR agreed on the detection of 10 positive samples, similar to the results from the full data set, and 853 negative samples (k = 0.26) (Table B in Supplementary Material). Two-by-two tables comparing test outcomes for the subset of 913 samples can be found in the Table B in Supplementary Material.

### Summary of herd testing data

ELISA classified 17% (28/159) of herds as positive based on 20 samples per herd and defined by one or more positive sample(s), compared to 6% (9/159) of herds that were classified as positive by pooled PCR out of all participating study herds (*N* = 159) (Table 3). ELISA and pooled PCR classified a similar proportion of positive herds when a positive herd was defined by two or more positive ELISA samples [5% (8/159)] (Table 3). The east had a higher proportion of test positive herds compared to the west by both pooled PCR and ELISA when a positive herd was defined by at least one positive pooled PCR sample or at least two positive ELISA samples (Table 3).

**Table 3:** Comparison of the proportion of MAP positive herds defined by either a 1 or more or 2 or more positive sample cut-off as well as the proportion of MAP positive samples within herds and the average within herd prevalence of MAP based on testing with serum ELISA and fecal PCR (pools of 5 and individual samples).

**Diagnostic test**	**Positive sample cut-off**	**Region**	**Total herds tested**	**Total test positive herds**	**Prevalence of test positive herds**	**Overall prevalence of test positive samples in positive herds**	**Within herd prevalence for test positive herds** ** (*****N*** = **159)**	
							**Mean**	**Min and max**
Serum ELISA	1 or more	West	110	19	0.17 (19/110)	0.07 (24/360)	0.06	0.05, 0.10
		East	49	9	0.18 (9/49)	0.09 (16/181)	0.09	0.05, 0.25
Serum ELISA	2 or more	West	110	5	0.05 (5/110)	0.10 (10/100)	0.10	0.10, 0.10
		East	49	3	0.06 (3/49)	0.17 (10/60)	0.17	0.10, 0.25
Fecal PCR (Pools of 5)	1 or more	West	110	5	0.05 (5/110)	0.10 (10/100)	0.10	0.05, 0.15
		East	49	4	0.08 (4/49)	0.25 (20/80)	0.25	0.05, 0.45

Of the 159 herds tested with pooled PCR and ELISA, 7 of the herds were positive by both pooled PCR and ELISA and 129 were negative on both (*k* = 0.32) when a positive herd consisted of at least one positive ELISA sample (Table 4). Pooled PCR and ELISA agreed on the detection of five positive herds and 147 negative herds (*k* = 0.57) when a positive herd consisted of at least two positive ELISA samples (Table 4).

**Table 4:** Comparison of MAP testing results for herds with one or more positive fecal PCR (pool of 5 fecal samples) result compared to herds with one or more positive ELISA result and two or more positive ELISA results for 159 beef herds.

	**One or more positive pooled PCR sample(s)**		
	**Positive**	**Negative**	**Total**
**One or more positive ELISA sample(s)**			
Positive	7	21	28
Negative	2	129	131
Total	9	150	159
Kappa = 0.32.			
	**Positive**	**Negative**	**Total**
**Two or more positive ELISA samples**			
Positive	5	3	8
Negative	4	147	151
Total	9	150	159

*Kappa = 0.57*.

ELISA also classified the highest proportion of herds as positive when defined by one or more positive sample(s) when comparing all three diagnostic tests (*N* = 128) (Table C in Supplementary Material). The agreement between pooled PCR and ELISA was similar when comparing results from the subset and full set of study herds (Table D in Supplementary Material; Table 4).

### Bayesian analysis

#### Test comparison at the animal level

**Figure 4:**
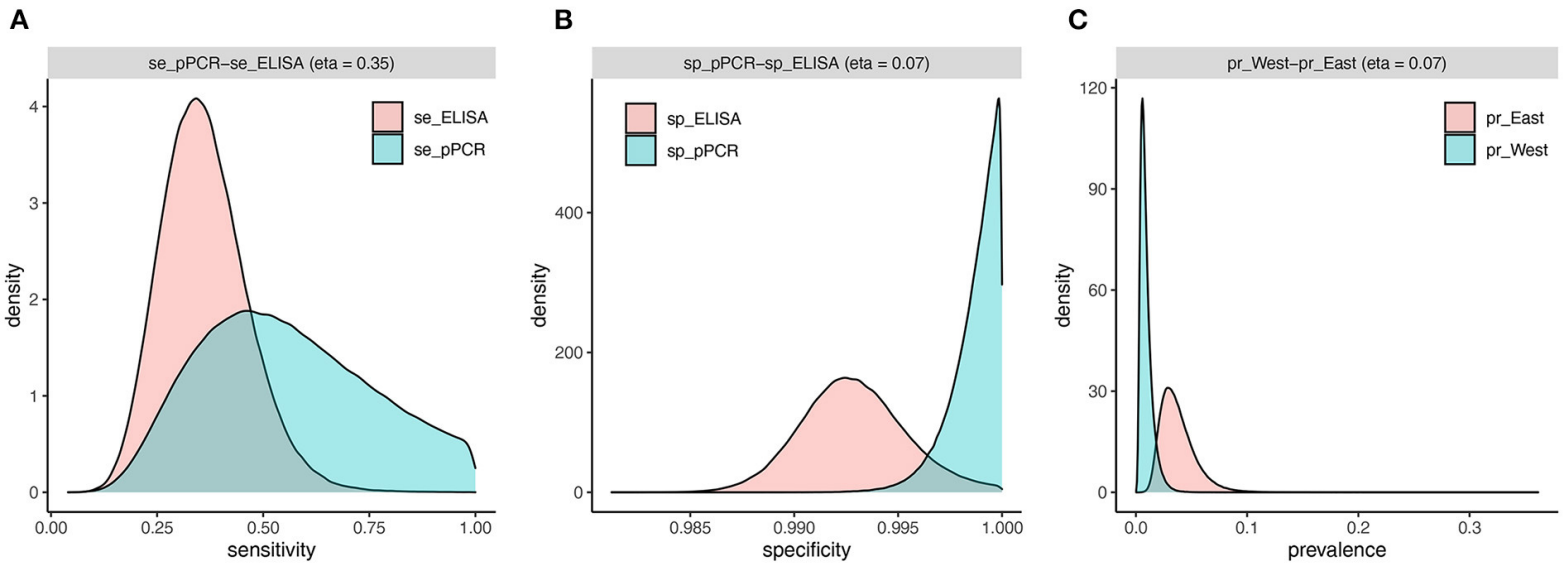
Estimated densities and overlap of posterior distributions from individual-level BLCM (*n* = 3,171 cows) for sensitivity **(A)** and specificity **(B)** of pooled PCR and ELISA, and prevalence **(C)** in the west and east regions.

The two-test comparison of ELISA and pooled PCR with uninformative priors using the full sample set (*n* = 3,171) yielded a higher median sensitivity for pooled PCR [54% (95% CrI 23–96%)] compared to ELISA [35% (95% CrI 17–56%)] (Table 5). The posterior distributions for sensitivity overlapped by 35% (Table 5; Figure 4) and the mean difference between posterior samples for the sensitivity of pooled PCR and ELISA was 0.19 (Table 5). Specificities were high for both tests at 99% (95% CrI 99–100%) for ELISA and 99.9% (95% CrI 99.6–100%) for pooled PCR (Table 5) with just 7% overlap of the posterior distributions (Table 5; Figure 4). The true disease prevalence was higher in the eastern region compared to the west (Table 5) with 7% overlap of the posterior distributions (Table 5; Figure 4).

**Table 5:** Sensitivity and specificity estimates for MAP diagnosis in 3,171 beef cows in the absence of a gold standard using a two-test, two population Bayesian latent class model with non-informative priors.

	**Median**	**95% Cr I**	**Proportion posterior distribution overlap** ^a^	**Mean difference in posterior estimates** ^b^
**Sensitivity**				
Pooled PCR	0.54	0.23, 0.96	0.35	0.19
ELISA	0.35	0.17, 0.56		
**Specificity**				
Pooled PCR	0.999	0.996, 1.00	0.07	0.006
ELISA	0.99	0.99, 1.00		
**Prevalence**				
West	0.01	0.002, 0.02	0.07	−0.03
East	0.03	0.01, 0.07		

a *Proportion of overlap between the posterior chains for estimates of parameters of interest from the BLCM where 0 reflects completely separate and 1 reflects completely overlapping distributions (49)*.

b *Mean of difference between pairs of posterior samples from the BLCMs (first parameter listed-second parameter)*.

The three-test comparison with uninformative priors at the animal level using the subset of samples (*n* = 913) tested with individual PCR yielded the highest sensitivity for individual PCR [96% (95% CrI 80–100%)] (Table 6). The posterior distribution for individual PCR sensitivity overlapped only 2% with that for pooled PCR and 0.4% with that for ELISA (Table 6; Figure A in Supplementary Material). Sensitivity and specificity estimates for pooled PCR and ELISA (Table 6) were very similar to the results from the two-test comparison using the full sample set (Table 5). The credible intervals for estimates of covariance parameters between Se and Sp for pooled and individual PCR included zero (Table 6), but were retained to ensure that dependence between these tests was accounted for in the model. The three-test comparison with informative priors yielded similar results to the comparison with uninformative priors (Table E in Supplementary Material).

**Table 6:** Sensitivity and specificity estimates for MAP diagnosis in 913 beef cows in the absence of a gold standard using a three-test, two population Bayesian latent class model with non-informative priors and using a subset of the data including only cows for which all three diagnostic tests were completed.

	**Median**	**95% CrI**	**Proportion posterior distribution overlap** ^a^	**Mean difference in posterior estimates** ^b^
**Sensitivity**				
Pooled PCR	0.54	0.36, 0.72		
Individual PCR	0.96	0.80, 1.00		
ELISA	0.36	0.22, 0.52		
Pooled PCR vs. Individual PCR			0.02	−0.40
Pooled PCR vs. ELISA			0.19	0.17
Individual PCR vs. ELISA			0.004	0.57
**Specificity**				
Pooled PCR	0.999	0.998, 1.00		
Individual PCR	0.98	0.96, 1.00		
ELISA	0.98	0.96, 0.99		
Pooled PCR vs. Individual PCR			0.03	0.02
Pooled PCR vs. ELISA			0.002	0.02
Individual PCR vs. ELISA			0.59	0.003
Prevalence^*^				
West	0.04	0.02, 0.06		
East	0.13	0.08, 0.17		
**Covariance**				
Se (individual/pooled)	0.00	−0.02, 0.04		
Sp (individual/pooled)	0.00	0.00, 0.00		

a *Proportion of overlap between the posterior chains for estimates of parameters of interest from the BLCM where 0 reflects completely separate and 1 reflects completely overlapping distributions (49)*.

b *Mean of difference between pairs of posterior samples from the BLCMs (first parameter listed-second parameter)*.

* *The prevalence reported here reflects a non-representative subsample of the overall data (913 of 3,171 total samples selected)*.

Two-test analyses were conducted between each of the three diagnostic tests using the subset of samples (*n* = 913) for comparative purposes (Table F in Supplementary Material). The results from the two-test analysis between pooled PCR and ELISA using the sample subset (Table F in Supplementary Material) were very similar to the results based on the full sample set (Table 5) suggesting no detectable impact of selection bias in the three-test comparison.

#### Test comparison at the pool level

A two-test comparison of PCR and ELISA with uninformative priors for detecting positive pools (*n* = 635) resulted in a higher median sensitivity for ELISA [76% (95% CrI 48–100%)] compared to PCR [44% (95% CrI 13–91%)] with 28% overlap in the posterior distributions (Table 7; Figure B in Supplementary Material). The estimated specificity of the PCR for classifying sample pools was 99.7% (95% CrI 99–100%) and for ELISA was 97% (95% CrI 95–100%) with 11% overlap in the posterior distributions (Table 7, Figure B in Supplementary Material).

**Table 7:** Sensitivity and specificity estimates for MAP diagnosis in 635 sample pools (5 fecal samples per pool) from 3,171 beef cows in the absence of a gold standard using a two-test, two population Bayesian latent class model with non-informative priors.

	**Median**	**95% CrI**	**Proportion posterior distribution overlap** ^a^	**Mean difference in posterior estimates** ^b^
**Sensitivity**				
Pooled PCR	0.44	0.13, 0.91	0.28	−0.28
ELISA	0.76	0.48, 1.00		
**Specificity**				
Pooled PCR	0.997	0.990, 1.00	0.11	0.02
ELISA	0.97	0.95, 1.00		
**Prevalence**				
West	0.03	0.00, 0.07	NA	NA
East	0.06	0.01, 0.13		

a *Proportion of overlap between the posterior chains for estimates of parameters of interest from the BLCM where 0 reflects completely separate and 1 reflects completely overlapping distributions (49)*.

b *Mean of difference between pairs of posterior samples from the BLCMs (first parameter listed-second parameter)*.

#### Test comparison at the herd level

In the two-test comparison of pooled PCR and ELISA with uninformative priors from the full set of study herds (*N* = 159) ELISA had a median sensitivity of 79% (95% CrI 47–100%) compared to pooled PCR at 43% (95% CrI 14–94%) when a positive herd consisted of at least one positive sample from 20 samples per herd (Table 8). Herd level specificity for ELISA was 90% (95% CrI 83–100%) and for pooled PCR was 99% (95% CrI 96–100%) (Table 8). Fewer herds were MAP positive in the west [10% (95% CrI 1–26%)] than in the eastern region [15% (95% CrI 2–35%)] although the posterior distributions overlapped by 60% (Table 8; Figure C in Supplementary Material).

**Table 8:** Sensitivity and specificity estimates for MAP diagnosis in 159 beef cow herds in the absence of a gold standard using two-test, two population Bayesian latent class models with non-informative priors.

	**Median**	**95% CrI**	**Proportion posterior distribution overlap** ^a^	**Mean difference in posterior estimates** ^b^
**Positive herd defined by one or more positive ELISA test**				
**Sensitivity**				
Pooled PCR (one or more +)	0.43	0.14, 0.94	0.28	−0.29
ELISA (one or more +)	0.79	0.47, 1.00		
**Specificity**				
Pooled PCR (one or more +)	0.99	0.96, 1.00	0.12	0.08
ELISA (one or more +)	0.90	0.83, 1.00		
**Prevalence**				
West	0.10	0.01, 0.26	0.60	−0.05
East	0.15	0.02, 0.35		
**Positive herd defined by two or more positive ELISA tests**				
**Sensitivity**				
Pooled PCR (one or more +)	0.73	0.37, 1.00	0.81	0.05
ELISA (two or more +)	0.67	0.32, 1.00		
**Specificity**				
Pooled PCR (one or more +)	0.98	0.95, 1.00	0.86	−0.003
ELISA (two or more +)	0.98	0.95, 1.00		
**Prevalence**				
West	0.05	0.00, 0.12	0.40	−0.05
East	0.09	0.02, 0.20		

a *Proportion of overlap between the posterior chains for estimates of parameters of interest from the BLCM where 0 reflects completely separate and 1 reflects completely overlapping distributions (49)*.

b *Mean of difference between pairs of posterior samples from the BLCMs (first parameter listed-second parameter)*.

Conversely, when a positive herd was defined by the detection of at least one positive pooled PCR sample and at least two positive ELISA samples from 20 samples per herd, pooled PCR had a sensitivity of 73% (95% CrI 37–100%) compared to ELISA at 67% (95% CrI 32–100%) with considerable (81%) overlap between the posterior distributions (Table 8; Figure D in Supplementary Material). The specificity for both tests was 98% (95% CrI 95–100%) with 86% overlap in the posterior distributions (Table 8, Figure D in Supplementary Material). Again, a lower disease prevalence was found in the west compared to the east although there was 40% overlap between the posterior distributions (Table 8, Figure D in Supplementary Material).

Two test analyses with uninformative priors were conducted between each of the three diagnostic tests using the subset of study herds (*N* = 128) for comparative purposes (Table G in Supplementary Material). The sensitivity and specificity estimates for pooled PCR and ELISA were similar between the full set of study herds (*N* = 159) and the subset of study herds (*N* = 128) suggesting minimal impacts of potential selection bias on estimates of sensitivity and specificity (Table 8; Table G in Supplementary Material). Convergence diagnostics, including potential scale reduction factor, effective sample size, Monte Carlo error, and visual inspection of trace and autocorrelation plots, were checked and suggested convergence criteria were met in all models.

### Associations between JD risk factors and positive test results

Based on the results from the univariable or unconditional analysis, animals with a BCS <2.5 were more likely (OR = 2.4, *p* = 0.03) to be ELISA positive compared to those with a BCS 2.5 (Table H in Supplementary Material). Furthermore, cows from herds that started calving in the summer or fall were more likely (OR = 5.2, *p* = 0.04) to test positive by ELISA compared to those from herds that calved in the winter (Table H in Supplementary Material). In the final multivariable model, only animals from herds that started calving in the summer or fall had a greater chance of being ELISA positive (OR = 5.26, *p* = 0.04) (Table 9).

**Table 9:** Summary of animal level multivariable regression analysis examining associations between potential risk factors and testing outcomes for 3,171 individual beef cows accounting for clustering within 159 herds using generalized estimating equations.

	**Odds ratio**	**95% CI**	***P*** **-value**
**Risk factors for a positive ELISA result**			
**Age**			
2–3 years	1 (base)		
>3 years	2.62	0.91–7.56	0.08
**BCS at pregnancy testing**			
≥2.5	1 (base)		
<2.5	2.27	0.97–5.32	0.06
**Season calving began**			
**Overall**			0.11
Winter	1 (base)		
Summer/fall	5.26	1.12–24.6	0.04^*^
Spring	1.37	0.56–3.33	0.49
**Risk factors for a positive pooled PCR result**			
**Calving location**			
Non-confined	1.00 (base)		
Confined	3.40	0.39–29.3	0.27
**Dairy cattle on farm**			
No	1.00 (base)		
Yes	9.74	1.40–67.9	0.02^*^
Number of females exposed to breeding	0.993	0.986–1.000	0.07

* *Significant at the p < 0.05 level*.

Region was associated with having a pooled PCR positive result, with animals from eastern herds being more likely (OR = 4.6, *p* = 0.046) to test positive compared to those from western herds in the unconditional models (Table I in Supplementary Material). Similarly, animals from herds that had dairy cattle on-farm were more likely (OR = 11.7, *p* = 0.02) to test positive by pooled PCR compared to those that were not (Table I in Supplementary Material). In the final multivariable model, only animals from herds with dairy cattle on-farm had a greater chance (OR = 9.7, *p* = 0.02) of testing positive with pooled PCR (Table 9).

Additional data were available for a subset of 2,150 cows from 108 of the participating herds. Cows from herds that had animals show symptoms of JD in the last 3 years were more likely to test positive by ELISA (OR = 4.6, *p* = 0.001) and pooled PCR (OR = 14, *p* = 0.02) compared to those that did not (Tables H, I in Supplementary Material). In the final multivariable analysis, cows from herds that had animals show symptoms of JD were more likely to test positive by ELISA (OR = 5.08, *p* = 0.0001) and pooled PCR (OR = 16, *p* = 0.01) (Table 10).

**Table 10:** Summary of animal level multivariable regression analysis examining associations between potential risk factors and testing outcomes from a subset of herds providing additional information: 2,150 individual beef cows accounting for clustering within 108 herds using generalized estimating equations.

		**Odds ratio**	**95% CI**	***P*** **-value**
**Risk factors for a positive ELISA result**				
Had animals show JD symptoms in last 3 years	No	1 (base)		
	Yes	5.08	2.04–12.6	0.0001^*^
Purchased replacements in last 5 years	Yes	1 (base)		
	No	2.28	0.89–5.82	0.09
**Risk factors for a positive pooled PCR result**				
Had animals show JD symptoms in last 3 years	No	1 (base)		
	Yes	16.8	1.99–141	0.01^*^
Purchased replacements in the last 5 years	Yes	1 (base)		
	No	4.22	0.68–26.3	0.12

* *Significant at the p < 0.05 level*.

There were no significant risk factors identified for testing positive by ELISA or pooled PCR across all 159 study herds based on the herd-level univariable analysis. For the subset of 108 herds for which additional data were available, herds that had animals show symptoms of JD in the last 3 years were more likely to test positive by ELISA (OR = 3.47, *p* = 0.03) and pooled PCR (OR = 16.6, *p* = 0.02) (Table 11).

**Table 11:** Summary of herd level multivariable regression analysis examining associations between potential risk factors and testing outcomes from a subset of 108 herds providing additional information.

		**Odds ratio**	**95% CI**	***P*** **-value**
**Risk factor for a positive ELISA result**				
Had animals show JD symptoms in last 3 years	No	1.00 (base)		
	Yes	3.47	1.11–10.9	0.03^*^
Purchased replacements in last 5 years	Yes	1.00 (base)		
	No	2.46	0.82–7.37	0.12
**Risk factor for a positive pooled PCR result**				
Had animals show JD symptoms in last 3 years	No	1 (base)		
	Yes	16.6	1.74–158.4	0.02^*^

* *Significant at the p < 0.05 level*.

## Discussion

The prevalence of MAP in beef cow-calf herds was higher in eastern Canada compared to western Canada at the animal and herd level based on both ELISA and PCR test results. Campbell et al. (56) reported similar findings in a study looking at the seroprevalence of MAP in Canadian cow-calf herds. This trend could be explained by the concentration of the dairy industry in eastern provinces and the associated risk due to a higher prevalence of MAP in dairy cattle (34, 57, 58). A difference in management practices between operations in the east and west could also account for the higher prevalence in the eastern region, as beef cattle are often raised more intensively in eastern Canada, potentially resulting in a greater risk of disease transmission (59).

In the present study, MAP ELISA seroprevalence in the western region was 1.1% at the animal level, 17% at the herd level with at least one positive sample per herd and 5% with at least two positive samples per herd defining a positive herd. These results were slightly higher than previously observed seroprevalences of 0.8% and 0.7% at the animal level, 15% and 20% at the herd level with at least one positive sample and 3% at the herd level with at least two positive samples reported in Saskatchewan beef herds on community pastures in 1999 (8) and a western Canadian study in 2001 (7), respectively.

A U.S. study conducted in 1997 estimating the prevalence of MAP in beef cow-calf herds from 21 states found an animal level seroprevalence of 0.4, and 7.9% of herds had at least one seropositive animal (60). The seroprevalence from this study was lower in comparison to those reported in Canadian studies from the same years. However, another study aimed at determining the prevalence of MAP in beef and dairy cull cattle located in Georgia using serum samples collected in 1999 found a prevalence of 3.9% in beef cattle and 9.6% in dairy cattle (61). The higher prevalence reported in this study could be due to the higher expected proportion of MAP positive animals among beef cattle that are culled due to age or poor productivity.

A study looking at MAP seroprevalence in Alberta cow-calf herds using samples collected during 2002–2003 reported a value of 1.5% at the animal level. At the herd level, a 28% seroprevalence was found for herds with at least one positive sample and 7.9% for herds with at least two positive samples (33). These seroprevalence values were slightly higher than those reported in the present study. However, the herds recruited to the Alberta study were limited to cattle in the herds serviced by the Johne's control program in Alberta at the time and were therefore more likely to be positive than the baseline population.

Campbell et al. (56) reported a seroprevalence of 0.8% at the animal level using samples collected from Canadian beef herds in 2003–2004. A more recent but smaller Alberta study of 840 cows from 28 Alberta ranches, also reported a seroprevalence of 0.8% from samples collected in 2011 (31). Results from MAP testing data collected through the Western Canadian Cow-calf Surveillance Network in 2014, the predecessor of the C^3^SN, found an animal level prevalence of 1.5% and a herd level prevalence of 5% when a positive herd consisted of at least two positive animals (62). These results are higher than those of the present study that were based on 2019 sampling data that included 42 of the same western herds that participated in the 2014 study. Of the 42 herds that provided data in both 2014 and 2019, the within herd prevalence of MAP increased in 6 herds, decreased in 8 herds and remained the same in 28 herds. The apparent decrease in prevalence observed in the western herds could be due in part to the herds acting on the realization there was JD in their herds from the 2014 testing, however, there were likely other factors that contributed to this decrease.

The results from the current study confirm that the prevalence of MAP in the Canadian cow-calf population remains low. However, the high prevalence of herds with at least one positive animal suggests that MAP presents an ongoing threat to the industry. Furthermore, the Canadian beef industry is becoming increasingly concentrated in the western part of the country and the number of animals per herd is growing (9). StatsCan Census data shows that from 2011 to 2016 the number of farms reporting beef cattle has decreased by 10.5% and the number of farms with 178 beef cows or more has increased by 9.2%. With ongoing consolidation of cow-calf herds, the risk of MAP spreading in the cow-calf industry will continue to increase.

Bayesian latent class models were used in this study to estimate the sensitivity and specificity of ELISA and PCR (individual and pooled) as well as the true prevalence of subclinical disease at both the animal and herd level. Overall, ELISA tended to have a lower specificity than pooled PCR at both the animal and herd level. However, when compared to specificity estimates from previous studies, the ELISA estimates at the animal level from the present study were among the highest. Due to the non-random selection of the sample subset that were tested with individual PCR there was the potential for bias. However, sensitivity and specificity estimates for ELISA and pooled PCR were very similar between the full set and subset of samples at the animal level suggesting the impact of any selection bias on estimates of test performance was not substantial.

Research on herd level estimates for diagnostic test sensitivity and specificity is lacking, especially in beef cattle, therefore the opportunity to make direct comparisons between past and present findings was limited. The herd level estimates reported here are limited by the sample size of 20 cows per herd. The interpretation of herd level estimates in the present study is specific to the capacity to detect a positive animal within a sample size of 20 rather than a determination of whether the herd is infected or not.

To the best of our knowledge, no previous studies have been published on diagnostic test performance for detecting MAP in Canadian beef cows or beef herds using the BLCM approach. The only other beef study referencing Bayesian methods was limited to estimating true prevalence based on a single ELISA test (31). However, several other studies have applied BLCM to examine test performance in dairy herds.

A study involving the use of BLCMs to estimate the sensitivity and specificity of serum ELISA and fecal culture in U.S. dairy cattle found a sensitivity of 60.6% in heavy shedding cattle and 18.7% in light shedding cattle for ELISA (25). The ELISA sensitivity estimate in the present study fell between the two values for heavy and light shedding cattle reported by Espejo et al. (25). The animals in the present study were not separated based on shedding intensity as the PCR provided a qualitative assessment of positive or negative. Espejo et al. (25) also reported a specificity value of 99.5% for ELISA, which was slightly higher than that of fecal culture (98.5%). These results are consistent with the estimates of specificity for ELISA (99%) and fecal PCR (100%) reported in the present study.

In another study evaluating test performance for MAP in dairy cows, the sensitivity and specificity of ELISA was estimated to be 41.4 and 97.7%, respectively when compared to fecal culture using BLCMs (26). Moreover, a study reporting on diagnostic test performance for MAP in Quebec dairy herds using the Bayesian estimation framework found a serum ELISA (IDEXX) sensitivity and specificity of 36.6 and 97.6%, respectively (27). Finally, a study conducted in Chilean dairy herds found a sensitivity and specificity of 26 and 98.5%, respectively for ELISA (IDEXX) when compared to fecal culture using Bayesian methods (28). The results from these previous studies are consistent with the sensitivity and specificity estimates reported in the present study.

A study using fecal culture as a gold-standard comparison to determine the sensitivity and specificity of ELISA (IDEXX) using samples from two Japanese dairy herds with a negative MAP status and three U.S. dairy herds with fecal culture MAP positive results found a sensitivity of 48.5% and specificity of 97.4% (52). The ELISA sensitivity estimate produced in this dairy study was higher than that produced in the present study while the dairy specificity estimate was lower in comparison. Another study using the fecal culture gold standard comparison approach found a sensitivity and specificity of 28.9 and 95.3%, respectively for detecting MAP in 14 U.S. dairy herds (51). These estimates were also lower than those found in the present study.

While the present study compared the performance of ELISA to that of PCR using BLCMs, most of the currently available published literature focused on the comparison of ELISA to fecal culture. Furthermore, previous studies using fecal culture as a gold standard focused mainly on estimating diagnostic test performance for detecting MAP in dairy cattle in which there is a higher disease prevalence. Despite these differences, most of the previous studies reported ELISA sensitivity and specificity estimates that were in a similar range to those produced in the present study using BLCMs (26–28, 52). Interestingly, the ELISA specificity estimate reported in this study was slightly higher than the estimates reported in most of the previous research (26–28, 51, 52).

Previous studies in the U.S. have reported that the exposure of beef cattle to environmental mycobacteria could contribute to the lower ELISA specificity reported in some older papers, as different species of mycobacteria had been shown to cross react with MAP antigens in serological tests resulting in false positives (63, 64). However, the findings from the present study suggest the current risk of false positive ELISA test results is more limited in the Canadian beef cattle population.

Combining fecal samples from multiple cows into pools for testing is a method of optimizing testing costs without sacrificing test performance (18). Few studies have been published on the performance of PCR on pools of individual fecal samples in beef cattle. A recent Australian study estimating the sensitivity and specificity of PCR for different pool sizes in beef cattle found that a pool size of 5 samples had a sensitivity of 63% and specificity of 100% (19). The present study found a lower sensitivity (44%) and similar specificity (99.7%) in comparison. A study evaluating the sensitivity of fecal culture and PCR on pools of samples with different dilution rates in dairy cattle found that PCR had a sensitivity of 83.8% for detecting a positive sample in pool sizes of five (65). This study reported a higher sensitivity in relation to the current study. A possible explanation for this could be that the positive fecal samples used in this dairy study were collected from animals that were shedding moderate to heavy levels of MAP resulting in higher detection rates. Test sensitivity estimates have been reportedly similar for both pool sizes of 5 and 10 and in some cases pool sizes of 10 have yielded higher sensitivities than a pool size of 5 in beef cattle herds (19, 66). Developing a highly sensitive pooled diagnostic test and testing strategy would improve the cost-effectiveness of screening herds and enhance control programs.

Another option for reducing testing costs is to consider risk-based testing. However, there is not a substantial amount of information on what risk factors might be best for selecting high risk animals within a cow-calf herd or for selecting high risk herds. In the unconditional analysis from this study, cows with a lower BCS were more likely to test positive for MAP by ELISA along with cows from herds that calved in the summer or fall and had animals show clinical signs of JD in the past 3 years. Furthermore, cows from eastern herds and cows from herds that had dairy cattle on the property were more likely to test positive for MAP by fecal PCR. A study looking at JD risk factors in purebred beef cattle in Texas found a greater chance of seropositivity when a dairy-type nurse cow was used, when clinical signs for JD had been previously recognized within the herd and with the use of seasonal calving in the spring (34). This study reports similar risk factors for testing positive for MAP compared to the present study.

While there is limited research on risk factors for JD at the animal level in beef herds there are several studies that have looked for herd level risk factors (31–33). Most studies to date have not had sufficient power to identify herd level risk factors for MAP infection in beef herds (31, 32). Scott et al. (33) were limited to looking at agro-ecological risk factors from available data linked to herds through geographic information systems. They did report regional differences in prevalence that could be related to differences in herd management whereby herds that were more likely to be part of traditional ranches had a lower seroprevalence than cow-calf herds that were part of more intensively managed farming operations.

Most of the work to look at risk factors to date that is available for comparison has been from dairy herds. A study conducted on risk factors associated with JD in Ontario dairy herds found that herds with a history of JD were more likely to have at least one seropositive animal as well as higher numbers of positive animals (67). These findings are in agreement with the present study that found herds were more likely to test positive by ELISA where animals had shown symptoms of JD in the previous 3 years. Furthermore, a study using environmental sampling to estimate the within-herd prevalence of MAP in western Canadian dairy herds reported greater odds of a herd testing positive if it consisted of more than 200 animals (68). The present study found that herds were more likely to test positive with an increasing number of breeding females.

Other herd level risk factors reported by previous studies in dairy were not supported by the present study. Tiwari et al. (58) found that farms with more than 200 acres of pasture were associated with fewer seropositive dairy cows compared to farms that had 100 acres of pasture or less. The present study subjectively assessed confinement in the calving area and found no significant association with likelihood of testing positive. Previous studies in dairy cattle reported an association between purchasing animals and herd positivity status with herds that had purchased animals within the past 12 months or 5 years being more likely to test positive (58, 67, 69). The current study did not find greater odds of testing positive in herds that purchased replacement animals in the past 5 years. Many of the differing results between past studies and the present study could be a reflection of the differences between beef and dairy industries.

The findings reported here should be viewed in the context of the practical limitations of this work. The surveillance network from which these herds were recruited were volunteers with a relationship with their veterinarian and who have at least some minimal herd records. While this convenience sample might not be completely representative of the Canadian industry, the method of recruitment is similar to that reported for many of the previous seroprevalence studies referenced from Canada. The relatively small number of samples testing positive and herds tested limits the power of the study to identify risk factors, and also resulted in wide confidence intervals for estimates of test sensitivities. Further, as these samples were collected as part of a sampling initiative within the network there was no JD specific concurrent survey of targeted risk factors at the time of sample collection. However, data from previous longitudinal surveys on these herds collected prior to sample collection were leveraged to fill in questions not asked at the time of sample collection. While this limited the risk factors we were able to investigate, it did provide more relevant time period-specific data than is typically available with many cross-sectional projects. Herd owners were contacted directly after sample collection to fill in some of the information that was not available in the existing database.

## Conclusion

The overall prevalence of Johne's disease in the Canadian cow-calf population remains low. The current estimate is that between 1 and 3% of cows are MAP positive and 10–15% of herds contain at least one animal that tested positive for MAP by either PCR or ELISA. These results indicate that JD continues to be a threat to the Canadian beef industry, especially with the ongoing consolidation of herds in western Canada. The results of this study suggest that ELISA could be an effective option for MAP screening at the herd level as the impact of lower sensitivity (36%) is mitigated to at least some extent by collecting multiple samples from each herd. Furthermore, the results found that the ELISA test had a relatively high specificity (98%) indicating that the risk of false positive test results is relatively low in this population. PCR was confirmed to be the more accurate method of identifying positive cases at the individual level. Therefore, initial testing with ELISA followed by pooled (Se = 54%, Sp = 99.9%) or individual (Se = 96%, Sp = 98%) animal fecal PCR could be a strategic option for identifying cases of JD in beef cattle within infected herds, but requires more study to examine cost-effectiveness and practical constraints.

## Data availability statement

The datasets presented in this article are not readily available because the terms of our agreement with participating producers preclude sharing of individual herd data. Key data are summarized in the Supplementary material. Requests to access the datasets should be directed to cheryl.waldner@usask.ca.

## Ethics statement

The animal study was reviewed and approved by the University Animal Care Committee Animal Research Ethics Board, University of Saskatchewan. Written informed consent was obtained from the owners for the participation of their animals in this study.

## Author contributions

PJ wrote the initial draft of the manuscript and coordinated manuscript editing with co-authors. PJ and CW prepared the dataset and performed descriptive and regression analyses. LM performed Bayesian latent class model analysis, participated in interpretation of results, and contributed to the writing of the manuscript. CW developed the study design, provided input on statistical analysis, participated in interpretation of results, and contributed to the writing of the manuscript. JC, KL, and MR edited the manuscript and provided feedback and revisions. All authors contributed to the article and approved the submitted version.

## Funding

This work was supported by the Saskatchewan Ministry of Agriculture (20180128), Beef Cattle Research Council (ANH.11.18), and NSERC (548206 – 18).

## Conflict of interest

The authors declare that the research was conducted in the absence of any commercial or financial relationships that could be construed as a potential conflict of interest.

## Publisher's note

All claims expressed in this article are solely those of the authors and do not necessarily represent those of their affiliated organizations, or those of the publisher, the editors and the reviewers. Any product that may be evaluated in this article, or claim that may be made by its manufacturer, is not guaranteed or endorsed by the publisher.

## Abbreviations

JD: Johne's disease
MAP: *Mycobacterium avium* subspecies *paratuberculosis*
Se: sensitivity
Sp: specificity
BLCM: Bayesian latent class models.
