# Supplementary material for: Estimating the sensitivity and specificity of serum ELISA and pooled and individual fecal PCR for detecting Mycobacterium avium subspecies paratuberculosis in Canadian cow-calf herds using Bayesian latent class models
Source: Front Vet Sci. 2022 Jul 29;9:937141. doi: 10.3389/fvets.2022.937141 (PMC9372466; doi:10.3389/fvets.2022.937141)
Supplement: Supplementary file 1 [file Data_Sheet_1.PDF]

Part I: Summary of data used to develop priors for Bayesian Latent Class Analysis to determine sensitivity and specificity for detection of MAP in the absence of a gold standard.

Table A. Sensitivity and specificity data from the literature used to develop informative priors for the Bayesian sensitivity analysis.

Diagnostic test	Sensitivity	Specificity	Author (date)
IDEXX ELISA	0.39 (27/70)	0.99 (271/274)	Whitlock et al (2000)
IDEXX ELISA	0.49 (33/68)	0.97 (37/38)	Scott et al (2010)
IDEXX ELISA	0.26 (6/23)	-	Speer et al (2006)
IDEXX ELISA	0.29 (120/415)	0.95 (342/359)	Collins et al (2005)
Real-time PCR Tetracore VetAlertTM kit	0.73	0.96	Alinovi et al. (2009)

Parameters for the informative beta prior distributions for ELISA were calculated from the literature as follows: alpha = x+1 and beta = n-x+1 where x was the number of successes and n was the number of tests summed across the relevant studies for each type of test. Resultant prior distributions were beta (187,391) for ELISA sensitivity and beta (651,22) for ELISA specificity. Estimates from the real-time PCR reference were used to develop priors using the EpiR beta buster function (55), using sensitivity and specificity estimates as the most likely values and conservative values for 95% lower bounds based on data presented (0.63 for sensitivity, 0.84 for specificity). Prior distributions for PCR were beta (48.33,18.51) and beta (27.71,2.11) for PCR sensitivity and specificity respectively.

Part II: Two by two tables comparing diagnostic test outcomes on a subset of 913 samples from beef cows tested for *Mycobacterium avium subsp. paratuberculosis* (MAP) using ELISA testing of serum samples, pooled PCR (5 cows per pool) and individual PCR testing of fecal samples.

Table B. Comparison of MAP testing results for ELISA testing of serum samples as compared to PCR testing of pools of five fecal samples or PCR testing of individual fecal samples for a subset of 913 samples* from beef cows examined with all three diagnostic tests.

		Pooled Fecal PCR		
		Positive	Negative	Total
ELISA Serology	Positive	10	30	40
	Negative	20	853	873
	Total	30	883	913
Kappa=0.26				

		Pooled Fecal PCR		
		Positive	Negative	Total
Individual Fecal PCR	Positive	30	42	72
	Negative	0	841	841
	Total	30	883	913
Kappa=0.57				

		Individual Fecal PCR		
		Positive	Negative	Total
ELISA Serology	Positive	19	21	40
	Negative	53	820	873
	Total	72	841	913
Kappa=0.30				

*See Figure 1 for a complete description of sample selection.

Part III: Comparison of diagnostic test outcomes on a subset of 128 beef herds screened for MAP using ELISA serology, and PCR testing of individual fecal samples as well as pools of five fecal samples.

Table C. Proportion of positive herds defined by one or more positive sample(s) and two or more positive samples as well as within herd prevalence detected by ELISA, pooled fecal PCR and individual fecal PCR (N=128)*.

Diagnostic test	Positive sample cut-off	Region	Total herds tested*	Total test positive herds	Prevalence of test positive herds	Overall prevalence of test positive samples in positive herds	Within herd prevalence for test positive herds(N=159)	
							Mean	Min and max
ELISA serology	1 or more	West	89	19	0.21 (19/89)	0.07 (24/372)	0.05	0.05, 0.10
		East	39	9	0.23 (9/39)	0.09 (16/176)	0.05	0.05, 0.25
ELISA serology	2 or more	West	89	5	0.06 (5/89)	0.10 (10/100)	0.10	0.10, 0.25
		East	39	3	0.08 (3/39)	0.17 (10/60)	0.15	0.10, 0.10
Fecal PCR (individual)	1 or more	West	89	12	0.13 (12/89)	0.17 (33/197)	0.19	0.05, 0.67
		East	39	7	0.18 (7/39)	0.28 (39/138)	0.28	0.05, 0.80
Fecal PCR (Pools of 5)	1 or more	West	89	5	0.06 (5/89)	0.10 (10/100)	0.10	0.05, 0.15
		East	39	4	0.10 (4/39)	0.25 (20/80)	0.25	0.05, 0.45

*See Figure 1 for a complete description of sample size per herd.

Table D. Comparison of MAP testing results for herds with at least one positive individual or pooled fecal PCR result compared to herds with at least one or at least two positive ELISA results for a subset of 128 beef herds examined with all three diagnostic tests.

		One or more positive pooled PCR sample(s)		
		Positive	Negative	Total
One or more positive individual PCR sample(s)	Positive	9	10	19
	Negative	0	109	109
	Total	9	119	128
Kappa=0.61				
		One or more positive individual PCR sample(s)		
		Positive	Negative	Total
One or more positive ELISA sample(s)	Positive	16	12	28
	Negative	3	97	100
	Total	19	109	128
Kappa=0.61				
		One or more positive individual PCR sample(s)		
		Positive	Negative	Total
Two or more positive ELISA samples	Positive	7	1	8
	Negative	12	108	120
	Total	19	109	128
Kappa=0.47				
		One or more pooled PCR sample(s)		
		Positive	Negative	Total
One or more positive ELISA sample(s)	Positive	7	21	28
	Negative	2	98	100
	Total	9	119	128
Kappa=0.30				
		One or more pooled PCR sample(s)		
		Positive	Negative	Total
Two or more positive ELISA sample(s)	Positive	5	3	8
	Negative	4	116	120
	Total	9	119	128
Kappa=0.56				

*See Figure 1 for a complete description of sample size per herd.

Part IV: Summary of supplemental results for Bayesian Latent Class Analysis to determine sensitivity and specificity for detection of MAP in the absence of a gold standard for a subset of 913 beef cows where three diagnostic tests were completed.

Table E. Sensitivity and specificity estimates for MAP diagnosis in beef cows in the absence of a gold standard using a three-test, two population Bayesian latent class model with informative priors developed using data from the literature and using a subset of the data including only cows for which all three diagnostic tests were completed (n=913 cows).

		Median	95% CrI
Sensitivity	Pooled Fecal PCR	0.68	0.58, 0.78
	Individual Fecal PCR	0.76	0.66, 0.84
	IDEXX ELISA for serum	0.32	0.29, 0.36
Specificity	Pooled Fecal PCR	0.98	0.97, 0.99
	Individual Fecal PCR	0.97	0.96, 0.99
	IDEXX ELISA for serum	0.97	0.96, 0.98
Prevalence*	West	0.04	0.02, 0.06
	East	0.14	0.09, 0.19
Covariance	Se (individual/pooled)	-0.02	-0.07, 0.02
	Sp (individual/pooled)	0.001	-0.001, 0.003

*Note the prevalence reported here reflects a non-representative subsample of the overall data (913 of 3171 total samples). See Figure 1 for description of sampling frame.

Table F. Sensitivity and specificity estimates for MAP diagnosis in beef cows in the absence of a gold standard using two-test, two population Bayesian latent class models with uninformative priors using a subset of the data including only cows for which three diagnostic tests were completed (n=913 cows).

		Pooled PCR vs. ELISA		Individual PCR vs. ELISA	
		Median	CrI	Median	CrI
Sensitivity	Pooled PCR	0.52	0.21, 0.94	-	-
	Individual PCR	-	-	0.72	0.46, 1.00
	ELISA	0.35	0.17, 0.55	0.30	0.17, 0.46
Specificity	Pooled PCR	0.996	0.99, 1.00	-	-
	Individual PCR	-	-	0.99	0.97, 1.00
	ELISA	0.97	0.96, 0.99	0.98	0.97, 1.00
Prevalence*	West	0.03	0.01, 0.08	0.06	0.02, 0.11
	East	0.12	0.05, 0.25	0.17	0.09, 0.29

*Note the prevalence reported here reflects a non-representative subsample of the overall data (913 of 3171 total samples). See Figure 1 for a description of the sampling frame.

Part V: Summary of supplemental results for Bayesian Latent Class Analysis to determine sensitivity and specificity for detection of MAP in the absence of a gold standard for a subset of 128 beef herds where three diagnostic tests were completed.

Table G. Sensitivity and specificity estimates for MAP diagnosis in beef cow herds in the absence of a gold standard using two-test, two population Bayesian latent class models with uninformative priors and a subset of the data where three diagnostic tests were completed (N=128 herds). This table is included only for comparison to analysis of the full data set of 159 herds to evaluate the potential impact of selection bias.

		Pooled PCR vs. 1 or more + ELISA		Pooled PCR vs. 2 or more + ELISA	
		Median	CrI	Median	CrI
Sensitivity	Pooled PCR	0.42	0.12, 0.93	0.73	0.36, 1.00
	ELISA	0.78	0.45, 1.00	0.67	0.31, 1.00
Specificity	Pooled PCR	0.98	0.94, 1.00	0.98	0.94, 1.00
	ELISA	0.87	0.78, 1.00	0.98	0.94, 1.00
Prevalence*	West	0.13	0.01, 0.32	0.06	0.004, 0.15
	East	0.20	0.03, 0.44	0.12	0.02, 0.25

*Note the prevalence reported here reflects a non-representative subsample of the overall data (913 of 3171 total samples and 128 of 159 herds). See Figure 1 for a description of the sampling frame.

Part VI: Summary of unconditional regression analysis examining associations between potential risk factors and testing outcomes for 3171 individual beef cows accounting for clustering within 159 herds using generalized estimating equations.

Table H. Summary of univariable logistic regression analysis of risk factors associated with a positive ELISA serology result for MAP in beef cows where P<0.20 (n=3171 cows from N=159 herds and n=2150 cows from N=108 herds).

Full set of 3171 cows from 159 herds				
Risk factor		Odds ratio	95% CI	P-value
Age	2-3 years	1 (base)		
	> 3 years	2.49	0.87 – 7.06	0.08
BCS at pregnancy testing	≥ 2.5	1 (base)		
	< 2.5	2.37	1.08 – 5.20	0.03*
Season calving began	Overall			0.11
	Winter	1 (base)		
	Summer/fall	5.17	1.11 – 24.0	0.04*
	Spring	1.40	0.58 – 3.40	0.45

Subset of 2150 cows from 108 herds				
Risk factor		Odds ratio	95% CI	P-value
Had animals show JD symptoms in last 3 years	No	1 (base)		
	Yes	4.62	1.86 – 11.5	0.001*
Purchased replacements in last 5 years	Yes	1 (base)		
	No	1.91	0.72 – 5.08	0.19

*Significant at the $p < 0.05$ level.

Table I. Summary of univariable logistic regression analysis of risk factors associated with a positive pooled fecal PCR result for MAP in beef cows where P<0.20 (n=3171 cows from N=159 herds and N=2150 cows from 108 herds).

Full set of 3171 cows from 159 herds				
Risk factor		Odds ratio	95% CI	P-value
Region	West	1 (base)		
	East	4.58	1.03 – 20.4	0.046*
Calving location	Non-confined	1 (base)		
	Confined	6.27	0.76 – 52.0	0.09
Season calving began	Overall			0.17
	Spring	1 (base)		
	Summer/fall	7.76	0.91 – 66.2	0.06
	Winter	1.80	0.30 – 10.7	0.52
Females exposed to breeding	Number	0.99	0.98 – 1.00	0.06
Dairy cattle on farm	No	1 (base)		
	Yes	11.7	1.41 – 97.2	0.02*

Subset of 2150 cows from 108 herds				
Risk factor		Odds ratio	95% CI	P-value
Had animals show JD symptoms in last 3 years	No	1 (base)		
	Yes	14.2	1.54 – 130.4	0.02*

*Significant at the $p < 0.05$ level.

Part VII: Comparison of densities of posterior distributions for individual and herd level results from BLCM analysis of MAP diagnostic tests with overlap analysis.

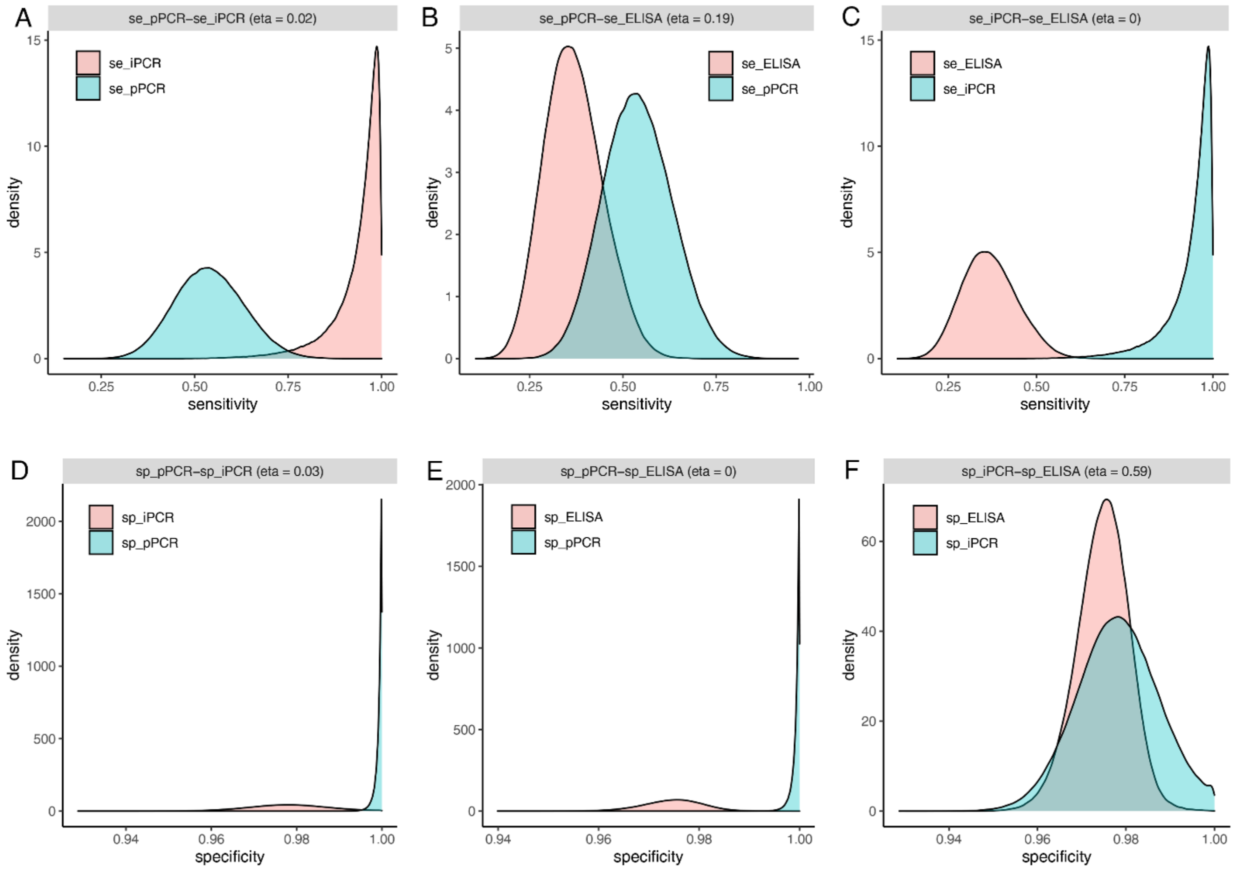

Figure A. Estimated densities and overlap of posterior distributions from individual-level BLCM (n=913 cows for which all three tests performed; uninformative priors) for sensitivity of pooled fecal PCR for MAP compared to individual PCR (A), sensitivity of pooled PCR compared to serum ELISA (B), sensitivity of individual PCR compared to ELISA (C), specificity of pooled PCR compared to individual PCR (D), specificity of pooled PCR compared to ELISA (E), and specificity of individual PCR compared to ELISA (F).

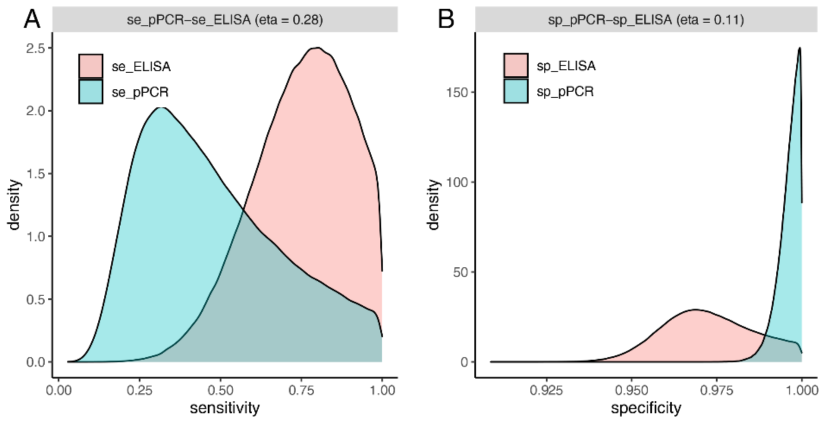

Figure B. Estimated densities and overlap of posterior distributions from pooled sample BLCM (n=635 pools of 5 samples) for sensitivity (A) and specificity (B) of pooled fecal PCR and serum ELISA for MAP.

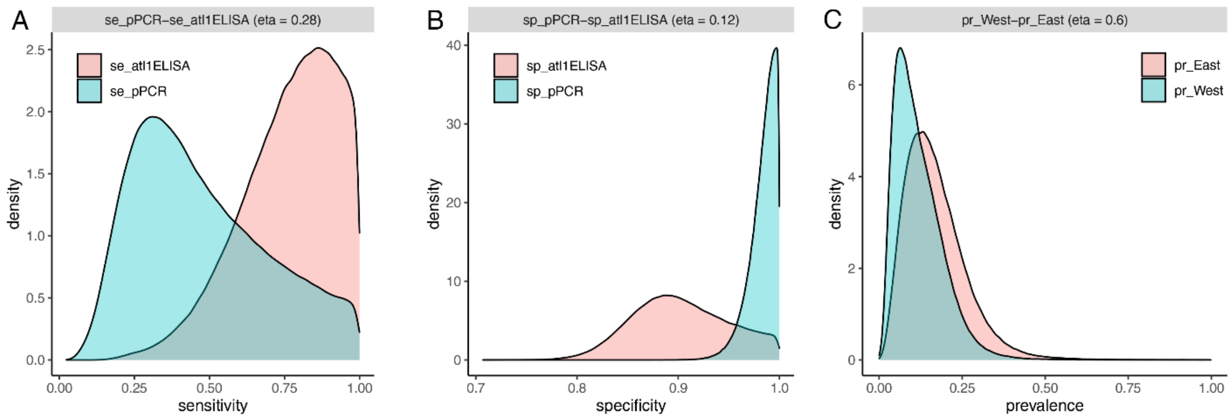

Figure C. Estimated densities and overlap of posterior distributions from herd-level BLCM (n=159 herds, 20 samples per herd) for sensitivity (A) and specificity (B) of one or more positive pooled fecal PCR and one or more positive serum ELISA for determining MAP positive herds, and prevalence (C) of positive herds in the west and east regions.

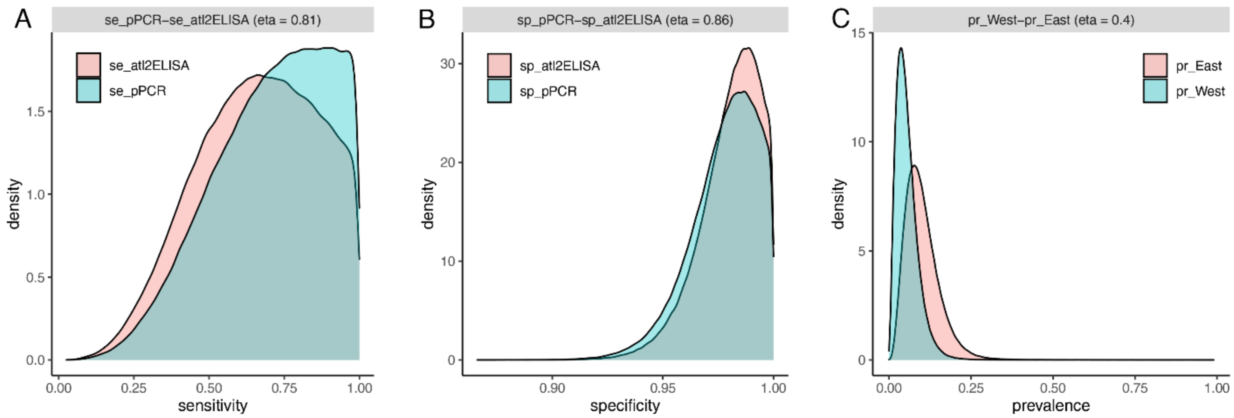

Figure D. Estimated densities and overlap of posterior distributions from herd-level BLCM (n=159 herds, 20 samples per herd) for sensitivity (A) and specificity (B) of one or more positive pooled fecal PCR and two or more positive serum ELISA for determining MAP positive herds, and prevalence (C) of positive herds in the west and east regions.

Part VIII: STARD-BLCM Checklist

Table J. The section or subsection in which each of the *Standards for the Reporting of Diagnostic accuracy studies that use Bayesian Latent Class Models* is addressed (adapted from http://www.equator-network.org/reporting-guidelines/stard-blcm/)

Section & Topic	No	Item	Reported on page #
**TITLE OR ABSTRACT**			
	1	Identification as a study of diagnostic accuracy, using at least one measure of accuracy (such as sensitivity, specificity, predictive values, or AUC) **and Bayesian latent class models**	Title
**ABSTRACT**			
	2	Structured summary of study design, methods, results, and conclusions (for specific guidance, see STARD for Abstracts)	Abstract
**INTRODUCTION**			
	3	Scientific and clinical background, including the intended use and clinical role of the **tests under evaluation**	Introduction
	4	Study objectives and hypotheses, **such as estimation of diagnostic accuracy of the tests for a defined purpose through BLCM**	Introduction, final paragraph
**METHODS**			
Study design	5	Whether data collection was planned before the **tests** were performed (prospective study) or after (retrospective study)	Sample collection
Participants	6	Eligibility criteria **and description of the source population**	Description and eligible study population
	7	On what basis potentially eligible participants were identified (such as symptoms, results from previous tests, inclusion in registry)	
	8	Where and when potentially eligible participants were identified (setting, location and dates)	
	9	Whether participants formed a consecutive, random or convenience series	Sample collection
Test methods	10	**Description of the tests under evaluation**, in sufficient detail to allow replication, **and/or cite references**	Serum samples, pooled fecal Samples, Individual fecal samples
	11	Rationale for choosing the **tests under evaluation in relation to their purpose**	Sample analysis
	12	Definition of and rationale for test positivity cut-offs or result categories of **the tests under evaluation**, distinguishing pre-specified from exploratory	Serum samples, Pooled fecal Samples, Individual fecal samples
	13	Whether clinical information was available to the performers or readers of **the tests under evaluation**	Sample analysis
Analysis	14a	**BLCM model** for estimating measures of diagnostic accuracy	Bayesian latent class models
	14b	**Definition and rationale of prior information and sensitivity analysis**	Informative priors and sensitivity analysis
	15	How indeterminate results **of the tests under evaluation** were handled	Data management and statistical analysis
	16	How missing data **of the tests under evaluation** were handled	Not applicable
	17	Any analyses of variability in diagnostic accuracy, distinguishing pre-specified from exploratory	Not applicable
	18	Intended sample size and how it was determined	Not applicable
**RESULTS**			
Participants	19	Flow of participants, using a diagram	Figure 1
	20	Baseline demographic and clinical characteristics of participants	Study herd characteristics
	21	**Not applicable: the distribution of the targeted conditions is unknown, hence the use of BLCM**	Not applicable
	22	Time interval and any clinical interventions between **the tests under evaluation**	Not applicable

Test results	**23**	Cross tabulation of the **tests' results (or for continuous tests results their distribution by infection stage)**	Tables 2 and 4 and B and D in supplement
	**24**	Estimates of diagnostic accuracy **under alternative prior specification** and their precision (such as 95% **credible/probability intervals**)	Table E (supplement)
	**25**	Any adverse events from performing **the tests under evaluation**	Not applicable
**DISCUSSION**			
	**26**	Study limitations, including sources of potential bias, statistical uncertainty, and generalisability	Final paragraph
	**27**	Implications for practice, including the intended use and clinical role of **the tests under evaluation in relevant settings (clinical, research, surveillance etc.)**	Throughout discussion
**OTHER**			
	**28**	Registration number and name of registry	Not applicable
	**29**	Where the full study protocol can be accessed	Not applicable
	**30**	Sources of funding and other support; role of funders	End of manuscript

Part IX: Sample Model Code

Two test – two population model

```
model{

	# Complete observations (N=3171):
	for(p in 1:Populations){
		Tally_RR[1:4,p] ~ dmulti(prob_RR[1:4,p], N_RR[p])

		prob_RR[1:4,p] <- se_prob[1:4,p] + sp_prob[1:4,p]
	}


	## Observation probabilities:

	for(p in 1:Populations){

		# Probability of observing test1- test2- from a true positive::
		se_prob[1,p] <- prev[p] * ((1-se[1])*(1-se[2]) +covse12)
		# Probability of observing test1- test2- from a true negative::
		sp_prob[1,p] <- (1-prev[p]) * (sp[1]*sp[2] +covsp12)

		# Probability of observing test1+ test2- from a true positive::
		se_prob[2,p] <- prev[p] * (se[1]*(1-se[2]) -covse12)
		# Probability of observing test1+ test2- from a true negative::
		sp_prob[2,p] <- (1-prev[p]) * ((1-sp[1])*sp[2] -covsp12)

		# Probability of observing test1- test2+ from a true positive::
		se_prob[3,p] <- prev[p] * ((1-se[1])*se[2] -covse12)
		# Probability of observing test1- test2+ from a true negative::
		sp_prob[3,p] <- (1-prev[p]) * (sp[1]*(1-sp[2]) -covsp12)

		# Probability of observing test1+ test2+ from a true positive::
		se_prob[4,p] <- prev[p] * (se[1]*se[2] +covse12)
		# Probability of observing test1+ test2+ from a true negative::
		sp_prob[4,p] <- (1-prev[p]) * ((1-sp[1])*(1-sp[2]) +covsp12)

	}


	## Priors:

	# Prevalence in population west:
	prev[1] ~ dbeta(1,1)

	# Prevalence in population east:
	prev[2] ~ dbeta(1,1)
```

```
        # Sensitivity of test1 test:
        se[1] ~ dbeta(1,1)T(1-sp[1], )
        # Specificity of test1 test:
        sp[1] ~ dbeta(1,1)

        # Sensitivity of test2 test:
        se[2] ~ dbeta(1,1)T(1-sp[2], )
        # Specificity of test2 test:
        sp[2] ~ dbeta(1,1)


        # Covariance in sensitivity between test1 and test2 tests:
        # covse12 ~ dunif( (se[1]-1)*(1-se[2]) , min(se[1],se[2]) - se[1]*se[2] )  ## if the sensitivity of
these tests may be correlated
         covse12 <- 0  ## if the sensitivity of these tests can be assumed to be independent
        # Covariance in specificity between test1 and test2 tests:
        # covsp12 ~ dunif( (sp[1]-1)*(1-sp[2]) , min(sp[1],sp[2]) - sp[1]*sp[2] )  ## if the specificity of
these tests may be correlated
         covsp12 <- 0  ## if the specificity of these tests can be assumed to be independent

}
```

Three test – two population model

```
model{
	# Complete observations (N=913):
	for(p in 1:Populations){
		Tally_RRR[1:8,p] ~ dmulti(prob_RRR[1:8,p], N_RRR[p])

		prob_RRR[1:8,p] <- se_prob[1:8,p] + sp_prob[1:8,p]
	}

	## Observation probabilities:
	for(p in 1:Populations){
		# Probability of observing test1- test2- test3- from a true positive::
		se_prob[1,p] <- prev[p] * ((1-se[1])*(1-se[2])*(1-se[3]) +covse12 +covse13 +covse23)
		# Probability of observing test1- test2- test3- from a true negative::
		sp_prob[1,p] <- (1-prev[p]) * (sp[1]*sp[2]*sp[3] +covsp12 +covsp13 +covsp23)

		# Probability of observing test1+ test2- test3- from a true positive::
		se_prob[2,p] <- prev[p] * (se[1]*(1-se[2])*(1-se[3]) -covse12 -covse13 +covse23)
		# Probability of observing test1+ test2- test3- from a true negative::
		sp_prob[2,p] <- (1-prev[p]) * ((1-sp[1])*sp[2]*sp[3] -covsp12 -covsp13 +covsp23)

		# Probability of observing test1- test2+ test3- from a true positive::
		se_prob[3,p] <- prev[p] * ((1-se[1])*se[2]*(1-se[3]) -covse12 +covse13 -covse23)
		# Probability of observing test1- test2+ test3- from a true negative::
		sp_prob[3,p] <- (1-prev[p]) * (sp[1]*(1-sp[2])*sp[3] -covsp12 +covsp13 -covsp23)

		# Probability of observing test1+ test2+ test3- from a true positive::
		se_prob[4,p] <- prev[p] * (se[1]*se[2]*(1-se[3]) +covse12 -covse13 -covse23)
		# Probability of observing test1+ test2+ test3- from a true negative::
		sp_prob[4,p] <- (1-prev[p]) * ((1-sp[1])*(1-sp[2])*sp[3] +covsp12 -covsp13 -covsp23)

		# Probability of observing test1- test2- test3+ from a true positive::
		se_prob[5,p] <- prev[p] * ((1-se[1])*(1-se[2])*se[3] +covse12 -covse13 -covse23)
		# Probability of observing test1- test2- test3+ from a true negative::
		sp_prob[5,p] <- (1-prev[p]) * (sp[1]*sp[2]*(1-sp[3]) +covsp12 -covsp13 -covsp23)

		# Probability of observing test1+ test2- test3+ from a true positive::
		se_prob[6,p] <- prev[p] * (se[1]*(1-se[2])*se[3] -covse12 +covse13 -covse23)
		# Probability of observing test1+ test2- test3+ from a true negative::
		sp_prob[6,p] <- (1-prev[p]) * ((1-sp[1])*sp[2]*(1-sp[3]) -covsp12 +covsp13 -covsp23)

		# Probability of observing test1- test2+ test3+ from a true positive::
		se_prob[7,p] <- prev[p] * ((1-se[1])*se[2]*se[3] -covse12 -covse13 +covse23)
		# Probability of observing test1- test2+ test3+ from a true negative::
		sp_prob[7,p] <- (1-prev[p]) * (sp[1]*(1-sp[2])*(1-sp[3]) -covsp12 -covsp13 +covsp23)

		# Probability of observing test1+ test2+ test3+ from a true positive::
```

```
        se_prob[8,p] <- prev[p] * (se[1]*se[2]*se[3] +covse12 +covse13 +covse23)
        # Probability of observing test1+ test2+ test3+ from a true negative::
        sp_prob[8,p] <- (1-prev[p]) * ((1-sp[1])*(1-sp[2])*(1-sp[3]) +covsp12 +covsp13 +covsp23)

    }

    ## Priors:

    # Prevalence in population west:
    prev[1] ~ dbeta(1,1)

    # Prevalence in population east:
    prev[2] ~ dbeta(1,1)


    # Sensitivity of test1 test:
    se[1] ~ dbeta(1,1)T(1-sp[1], )
    # Specificity of test1 test:
    sp[1] ~ dbeta(1,1)

    # Sensitivity of test2 test:
    se[2] ~ dbeta(1,1)T(1-sp[2], )
    # Specificity of test2 test:
    sp[2] ~ dbeta(1,1)

    # Sensitivity of test3 test:
    se[3] ~ dbeta(1,1)T(1-sp[3], )
    # Specificity of test3 test:
    sp[3] ~ dbeta(1,1)


    # Covariance in sensitivity between test1 and test2 tests:
    covse12 ~ dunif( (se[1]-1)*(1-se[2]) , min(se[1],se[2]) - se[1]*se[2] )  ## if the sensitivity of these
tests may be correlated
    # covse12 <- 0  ## if the sensitivity of these tests can be assumed to be independent
    # Covariance in specificity between test1 and test2 tests:
    covsp12 ~ dunif( (sp[1]-1)*(1-sp[2]) , min(sp[1],sp[2]) - sp[1]*sp[2] )  ## if the specificity of these
tests may be correlated
    # covsp12 <- 0  ## if the specificity of these tests can be assumed to be independent

    # Covariance in sensitivity between test1 and test3 tests:
    # covse13 ~ dunif( (se[1]-1)*(1-se[3]) , min(se[1],se[3]) - se[1]*se[3] )  ## if the sensitivity of
these tests may be correlated
     covse13 <- 0  ## if the sensitivity of these tests can be assumed to be independent
    # Covariance in specificity between test1 and test3 tests:
    # covsp13 ~ dunif( (sp[1]-1)*(1-sp[3]) , min(sp[1],sp[3]) - sp[1]*sp[3] )  ## if the specificity of
these tests may be correlated
     covsp13 <- 0  ## if the specificity of these tests can be assumed to be independent
```

```
	# Covariance in sensitivity between test2 and test3 tests:
	# covse23 ~ dunif( (se[2]-1)*(1-se[3]) , min(se[2],se[3]) - se[2]*se[3] )  ## if the sensitivity of these tests may be correlated
	 covse23 <- 0  ## if the sensitivity of these tests can be assumed to be independent
	# Covariance in specificity between test2 and test3 tests:
	# covsp23 ~ dunif( (sp[2]-1)*(1-sp[3]) , min(sp[2],sp[3]) - sp[2]*sp[3] )  ## if the specificity of these tests may be correlated
	 covsp23 <- 0  ## if the specificity of these tests can be assumed to be independent

}
```
